# Tunable Magnetism and Intrinsic Exchange Bias in Al‐Substituted Terbium Iron Garnet

**DOI:** 10.1002/adma.202510669

**Published:** 2025-09-18

**Authors:** Takayuki Shiino, Matteo Fettizio, Saúl Estandía, Can Onur Avci

**Affiliations:** ^1^ Institut de Ciència de Materials de Barcelona (ICMAB‐CSIC) Campus de la UAB Bellaterra 08193 Spain

**Keywords:** exchange bias, ferrimagnetism, magnetic compensation, rare‐earth iron garnet, spintronics

## Abstract

Ferrimagnetic insulators are central to both fundamental magnetism and diverse technologies, including spintronics, photonics, and microwave engineering. Their low damping, electrical insulation, and tunable magnetism make them ideal, especially for low‐power spintronic devices. Controlling key magnetic properties —particularly the magnetic compensation— is essential for accessing ultrafast dynamics, and advanced spintronic functionalities. Here, it is demonstrated that the magnetic compensation temperature (*T*_M_) of an archetype ferrimagnetic insulator, terbium iron garnet (Tb_3_Fe_5_O_12_, TbIG), can be continuously tuned and raised to ambient temperature by partially substituting magnetic Fe atoms with nonmagnetic Al. This substitution, achieved by high‐temperature co‐sputtering of TbIG and Al_2_O_3_, is confirmed by atomically resolved electron microscopy. Near *T*_M_, a giant intrinsic exchange bias of up to 2.5 kOe is observed. The exchange bias exhibits deterministic or stochastic behavior depending on the cooling conditions, and its polarity can be controlled via an external magnetic field. To explain the observed phenomena, a phenomenological model is developed that takes into account a distribution of local *T*_M_ values induced by magnetic site disorder. These findings provide an efficient strategy for controlling *T*_M_ and enabling exchange bias in TbIG that may add new functionalities for room‐temperature spintronic and photonic applications.

## Graphical abstract

Al substitution in terbium iron garnet thin films enables continuous tuning of the magnetic compensation temperature from 190 K to above room temperature. Near compensation, a giant intrinsic exchange bias emerges, whose sign and stochasticity can be controlled by external fields and cooling history. This approach provides new routes to engineer garnet thin films for spintronic applications.

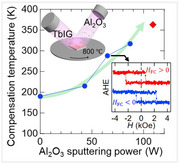

## 1 Introduction

Ferrimagnetism is a type of magnetic ordering found in materials where magnetic moments on different sublattices are antiferromagnetically coupled with unequal magnitudes, resulting in a net spontaneous magnetization.^[^ ^1^ ^]^ The coupling between these sublattices can lead to diverse magnetic behaviors such as noncollinear ordering, magnetic frustration, and magnetic compensation, where the net moment vanishes despite the presence of ordered magnetic moments.^[^ ^1^, ^2^ ^]^ Near the magnetic compensation temperature (*T*_M_) and its associated angular‐momentum compensation temperature,^[^ ^3^, ^4^ ^]^ intriguing magnetic and spintronic phenomena have been predicted and observed, such as ultrafast domain wall dynamics,^[^ ^5^, ^6^ ^]^ efficient spin‐orbit torques,^[^ ^7^, ^8^ ^]^ strong magnon–magnon coupling,^[^ ^9^ ^]^ and spin canting.^[^ ^10^ ^]^

Among ferrimagnets, rare‐earth iron garnets (REIG), with the chemical formula RE_3_Fe_5_O_12_ (where RE is a rare‐earth element), are of particular interest for insulator spintronics, owing to their favorable magnetic properties and compatibility with magnon‐ and spin‐based devices.^[^ ^3^, ^11^, ^12^, ^13^ ^]^ The magnetism in REIGs is dictated by the interplay of three distinct magnetic sites —*a*‐, *d*‐, and *c*‐sites— coordinated by oxygen atoms in octahedral, tetrahedral, and dodecahedral arrangements, respectively.^[^ ^2^ ^]^ The Fe^3 +^ moments on the *a*‐ and *d*‐sites are antiferromagnetically coupled via superexchange interactions and determine the magnetic ordering temperature *T*_c_ (approximately 560 K in bulk). RE^3 +^ moments in the *c*‐sites are antiferromagnetically coupled to *d*‐site Fe^3 +^ moments, ultimately leading to magnetic compensation. In their stoichiometric form, *T*_M_ of REIGs, if present, is typically below room temperature and the highest values are found in Gd_3_Fe_5_O_12_ (GdIG, *T*_M_ = 286 K) and Tb_3_Fe_5_O_12_ (TbIG, *T*_M_ = 246 K).^[^ ^14^ ^]^ These *T*_M_ values and other magnetic properties can be tuned by partially substituting RE or Fe with other elements. However, the difficulty of incorporating substitutes into the intricate garnet structure combined with the difficulty of growing high‐quality single crystals makes it challenging to precisely control magnetic parameters in REIGs, especially bringing *T*_M_ around room temperature, critical for spintronic applications. This limitation contrasts with metallic ferrimagnetic alloys such as GdFeCo^[^ ^15^ ^]^ and GdCo,^[^ ^6^ ^]^ in which *T*_M_ can be continuously and flexibly tuned by composition adjustments via co‐deposition.^[^ ^5^, ^6^, ^16^ ^]^ Near or above room temperature *T*_M_ has been reported before for some REIG thin films. Rosenberg et al. have reported Tb‐rich TbIG with antisite and vacancy defects with a *T*_M_ of 335 K,^[^ ^17^, ^18^ ^]^ nearly 90 K above that of stoichiometric TbIG. Similarly, a significant *T*_M_ increase has been observed in TbIG^[^ ^19^ ^]^ and GdIG^[^ ^20^ ^]^ films when their thickness is below 10 nm, which is likely associated with site disorder and intermixing near the substrate interface.^[^ ^19^, ^21^ ^]^ Nonetheless, these findings do not represent a systematic approach but rather happen to be consequences of a specific deposition condition or thickness choice. Therefore, precise user control over *T*_M_ similar to metallic ferrimagnets, especially near room temperature, in a technologically relevant REIG is still elusive.

**Figure 1:**
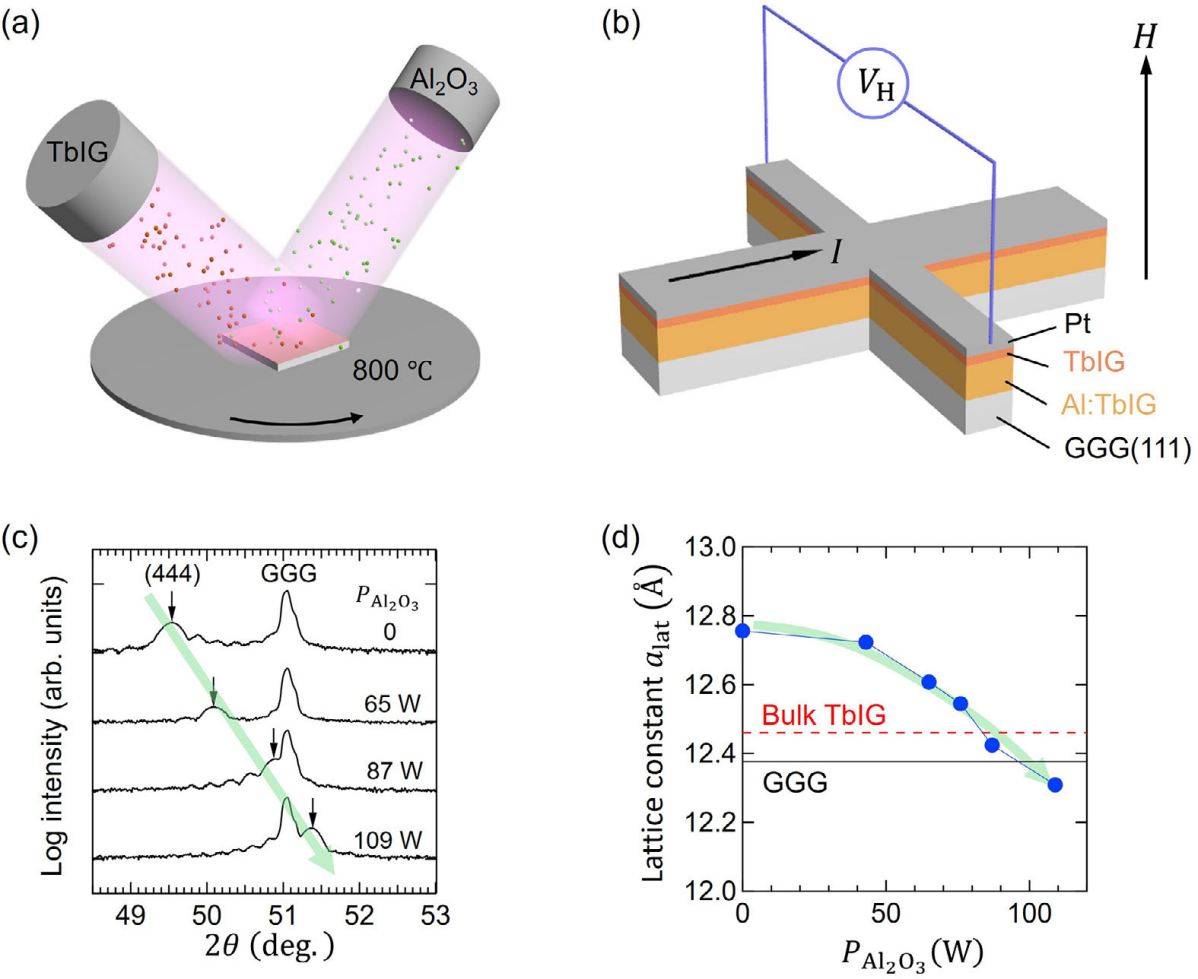
a) Schematic of the co‐sputtering process used to deposit TbIG and Al_2_O_3_ onto GGG(111) substrate. b) Schematic of the Hall‐bar device structure used for magnetotransport measurements. c) X‐ray diffraction (XRD) patterns of films with varying $P_{{\mathrm{Al}}_{2}O_{3}}$ showing a shift of the (444) TbIG peak with increasing Al content. d) Extracted lattice constant (*a*_lat_) as a function of $P_{{\mathrm{Al}}_{2}O_{3}}$, indicating lattice contraction due to Al substitution. The horizontal black line indicates the lattice constant of the GGG substrate, whereas the horizontal red dashed line represents the lattice constant of bulk TbIG.

Here, we control *T*_M_ of TbIG by using Al doping as a means to reduce the Fe contribution to magnetism. Doping is realized by co‐sputtering of TbIG and alumina (Al_2_O_3_) onto Gd_3_Ga_5_O_12_ [GGG(111)] substrates [see **Figure** **1**a]. Al replaces Fe in the garnet structure, which we confirm using transmission electron microscopy and spectroscopy. We systematically control *T*_M_ of TbIG in the 190–350 K temperature range by adjusting the amount of Al. Furthermore, we observe an unexpected and gigantic intrinsic exchange bias effect in the vicinity of *T*_M_ with a stochastic or deterministic nature depending on the field and temperature history of the device under test. We develop a phenomenological model to explain the origin and stochasticity of the exchange bias effect near *T*_M_ in the presence of disorder, considering a statistical distribution of local *T*_M_ in the film. Our work introduces an effective method to tune the *T*_M_ of REIGs and the intrinsic exchange bias as an additional functionality in REIG‐based spintronics.

## 2 Results

### 2.1 Layer Growth

We grew Al_2_O_3_ and TbIG simultaneously onto a GGG(111) substrate by magnetron co‐sputtering [Figure 1a] to obtain 23‐nm‐thick Al‐doped TbIG (denoted Al:TbIG hereafter). The relative concentration of Al_2_O_3_ and TbIG deposited onto the GGG surface was controlled by sputtering power of the respective targets, which was varied for the former between 0 – 109 W and kept fixed at 150 W for the latter. Based on prior independent calibration of the deposition rates, we estimate the Al content to vary between 0 – 8% in the above power range [see Section S1, Supporting Information]. However, for the sake of consistency, we refer to Al_2_O_3_ sputtering power ($P_{{\mathrm{Al}}_{2}O_{3}}$) to discuss layers with different Al concentration. The resulting Al:TbIG was then subsequently capped with a 2 nm‐thick TbIG and 4 nm‐thick Pt without breaking the vacuum. The full heterostructure Pt(4 nm)/TbIG(2 nm)/Al:TbIG(≈23 nm)//GGG was then fabricated into Hall bars by optical lithography and reactive ion etching [Figure 1b]. TbIG and Al:TbIG (up to a certain Al concentration) grow with a compressive strain onto GGG(111), leading to perpendicular magnetic anisotropy (PMA),^[^ ^22^, ^23^ ^]^ which we confirm by anomalous Hall effect (AHE) and polar Kerr effect (not shown) measurements. In the Hall bar device, electrical current flows only in the Pt layer and gives rise to a magnetization‐dependent Hall voltage (*V*_H_) stemming from the transverse component of the spin Hall magnetoresistance.^[^ ^24^ ^]^ Note that, in this work, we plot the Hall resistance (*R*_H_), obtained by normalizing the measured *V*_H_ with the applied current. In this study, we use an out‐of‐plane field sweep to detect the perpendicular component of the magnetization in Al:TbIG through the AHE component of the spin Hall magnetoresistance in Pt.^[^ ^25^ ^]^ We note that the 2‐nm TbIG was inserted to improve the interfacial spin transparency necessary for the spin Hall magnetoresistance to occur, hence the AHE signal, while the intrinsic exchange bias was observed even when this layer was omitted, i.e., Pt was grown directly onto Al:TbIG (see Section S2, Supporting Information). Further details are provided in Experimental Section.

### 2.2 Structural Characterization

We first discuss the influence of $P_{{\mathrm{Al}}_{2}O_{3}}$ on the structural and magnetic properties of Al:TbIG. Figure 1c presents the x‐ray diffraction (XRD) patterns in 2θ − ω geometry for the co‐sputtered film with different $P_{{\mathrm{Al}}_{2}O_{3}}$. The (444) TbIG peak appears at 49.5 ° as customary in such films,^[^ ^23^ ^]^ and it progressively shifts toward higher angles as $P_{{\mathrm{Al}}_{2}O_{3}}$ increases. At $P_{{\mathrm{Al}}_{2}O_{3}}$ = 109 W, the film transits from compressive to tensile strain as the (444) peak crosses over to the right side of the GGG substrate peak. Based on the XRD data and under the assumption that the cubic unit cell of TbIG is preserved, we estimated the lattice constant (*a*_lat_) of Al:TbIG as shown in Figure 1d. The lattice constant *a*_lat_ systematically decreases with the incorporation of Al. This behavior is also reflected in the magnetic properties of the film, which exhibits PMA for $P_{{\mathrm{Al}}_{2}O_{3}}<100$ W and mixed anisotropy (weak PMA combined with in‐plane anisotropy) for $P_{{\mathrm{Al}}_{2}O_{3}}>100$ W (see Section S3, Supporting Information). The structural and magnetic changes are attributed to the partial substitution of Fe in the TbIG phase by Al, creating a chemical pressure effect and reduced contribution of Fe to the net magnetization. Note that the observed nonlinear dependence of the lattice parameter on $P_{{\mathrm{Al}}_{2}O_{3}}$ may result from a combination of interfacial strain effects and bulk chemical pressure effects. The Al–Fe substitution is confirmed by electron spectroscopy as discussed later. Our observation aligns with previous studies reporting a reduction in the value of *a*_lat_ due to Fe–Al substitution in YIG.^[^ ^26^, ^27^, ^28^ ^]^ We note that for the highest $P_{{\mathrm{Al}}_{2}O_{3}}$ (= 109 W), an AlO_*x*_ impurity phase was clearly observed in the XRD data (see Section S4, Supporting Information), evidencing the formation of AlO_*x*_ precipitates within the film instead of a homogeneous distribution.

**Figure 2:**
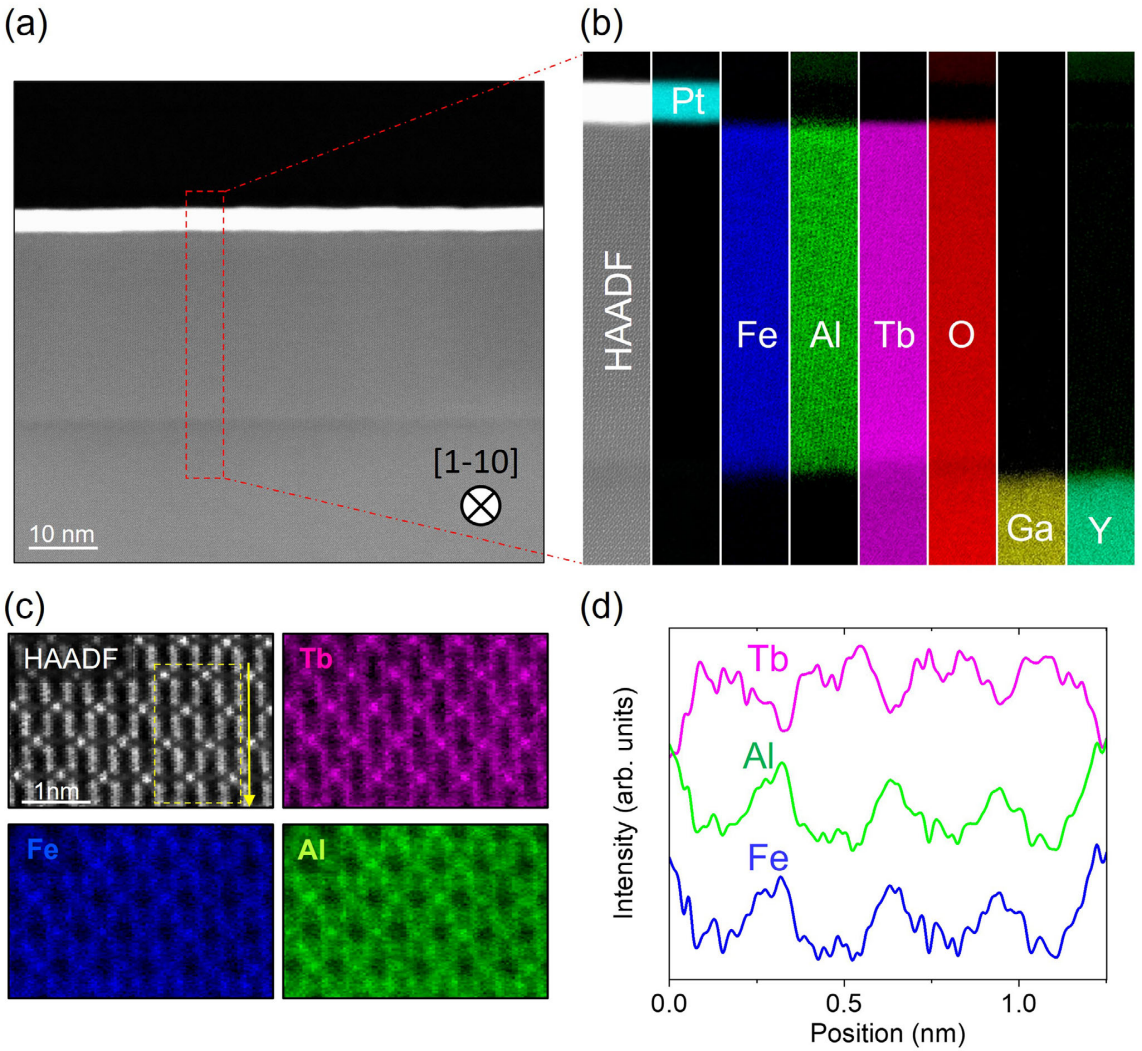
a) Low magnification HAADF‐STEM image of the full heterostructure cross‐section. b) EELS elemental maps showing the spatial distribution of the elements calculated from the corresponding edges (Pt‐M, Fe‐L, Al‐K, Tb‐M, O‐K, Ga‐L, and Y‐L). c) High‐resolution EELS maps and HAADF image highlighting the distribution of Tb, Fe, and Al. d) Line profiles of EELS intensity along the yellow arrow in (c), showing in‐phase Fe and Al signals, indicating Al substitution at Fe sites.

Next, we performed scanning transmission electron microscopy (STEM) and electron energy loss spectroscopy (EELS) to analyze the film crystalline structure with atomic resolution. For these measurements, we used Al:TbIG with $P_{{\mathrm{Al}}_{2}O_{3}}$ = 65 W grown on a Y‐doped terbium gallium garnet (TGG) substrate, which is a variant of GGG. This thin film was deposited simultaneously with the main sample where we studied the exchange bias effect, discussed later. **Figure** **2**a displays the low‐magnification high‐angle annular dark‐field (HAADF) survey image of the cross section of the film, confirming the uniformity of each layer constituting the heterostructure. Figure 2b presents the EELS elemental maps of atomic species contained in the sample corresponding to the red dashed box in Figure 2a. These maps show overall excellent homogeneity of all atoms in their corresponding layers and sharp interfaces between Al:TbIG and the substrate (bottom) and Pt (top). Interestingly, Al is also present in the topmost region of the garnet structure with a concentration gradient (see Section S5, Supporting Information), which was terminated by a 2 nm‐thick TbIG (without Al_2_O_3_). This indicates that Al tends to diffuse into the top TbIG region due to the high‐temperature growth and to minimize the lattice mismatch at the Al:TbIG and TbIG interface. We also note that the Al concentration estimated in Section S1 (Supporting Information) is compatible with our crude estimate from the EELS data (see Section S5, Supporting Information).

Figure 2c presents a high‐magnification HAADF image and EELS maps for Tb, Fe, and Al elements viewed along the [1‐10] direction. We note that the HAADF and EELS maps have been geometrically corrected (see Section S5, Supporting Information). The EELS profile along the arrow direction is plotted in Figure 2d. The profiles of Fe and Al are in‐phase, contrary to the out‐of‐phase Tb profile, clearly showing that Al occupies Fe sites in the garnet structure.

### 2.3 Influence of Al Doping on Magnetic Ordering Temperatures

**Figure 3:**
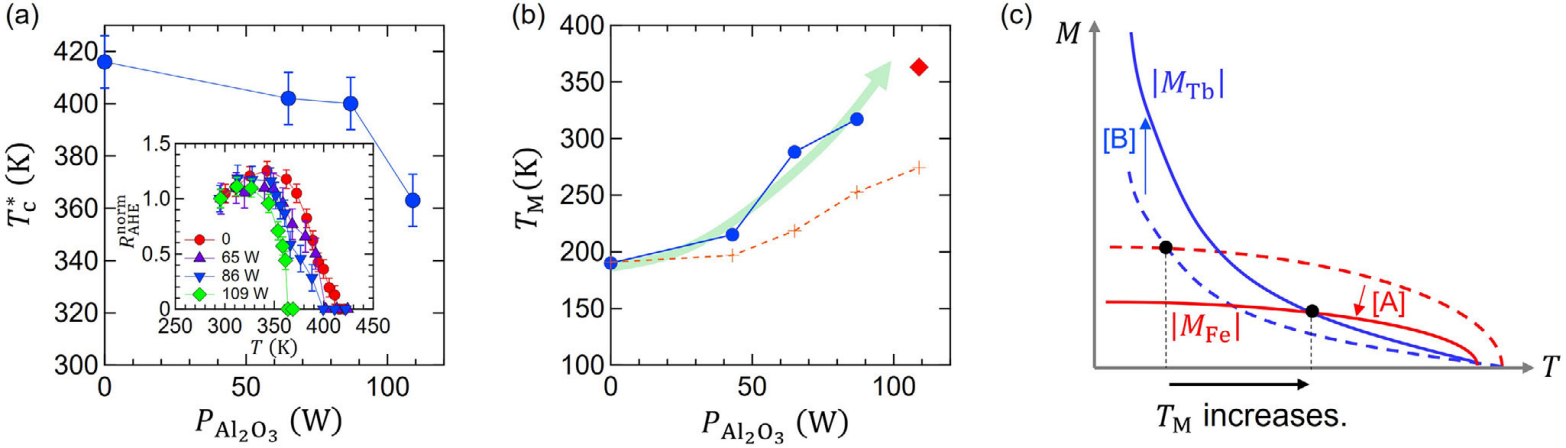
Variations of a) the estimated Curie temperature ($T_{c}^{*}$), and b) the magnetic compensation temperature (*T*_M_) plotted as functions of $P_{{\mathrm{Al}}_{2}O_{3}}$. The inset in (a) shows the temperature dependence of the normalized AHE resistance $R_{\mathrm{AHE}}^{\mathrm{norm}}\equiv R_{\mathrm{AHE}}\left(T\right)/R_{\mathrm{AHE}}\left(297\;K\right)$ for $P_{{\mathrm{Al}}_{2}O_{3}}=$ 0, 65, 86, and 109 W. The red diamond symbol in (b) indicates the value of $T_{c}^{*}$ for $P_{{\mathrm{Al}}_{2}O_{3}}=109$ W since the magnetic compensation was not reached until the ordering temperature in this film. The orange cross symbols connected by a broken line in (b) indicate the estimated values of *T*_M_ arising solely from the reduction in the lattice constant. c) Schematic illustration of the sublattice magnetizations of TbIG, showing the magnitudes of *M*_Tb_ and *M*_Fe_ (plotted as |*M*_Tb_| and |*M*_Fe_|). The broken curves represent the undoped systems, whereas the solid curves correspond to the Al‐substituted systems. The intersection point of |*M*_Tb_| and |*M*_Fe_| indicates the compensation temperature, *T*_M_. The red arrow (labeled [A]) highlights the reduction of *M*_Fe_ due to Al substitution, while the blue arrow (labeled [B]) indicates the enhancement of *M*_Tb_ resulting from the lattice contraction induced by Al substitution.

We leverage AHE measurements in Pt overlayers to infer the magnetic properties of Al:TbIG films. We first characterize the ordering (Curie) temperature ($T_{c}^{*}$) by measuring the AHE as a function of temperature. Assuming that the spin Hall effect in Pt does not strongly depend on temperature,^[^ ^29^ ^]^ we hypothesize that the AHE should vanish when the film loses its magnetic ordering.^[^ ^30^ ^]^ Therefore, it is an indirect measure of the ordering temperature, hence the asterisk on *T*_c_. **Figure** **3**a shows $T_{c}^{*}$ as a function of $P_{{\mathrm{Al}}_{2}O_{3}}$ based on the AHE measurements reported in the inset. We find that $T_{c}^{*}$ is slightly above 400 K in the film without Al and gradually decreases as the Al content increases. This is an expected result since *T*_c_ in REIGs is dictated by Fe‐containing magnetic sublattices with little influence on the RE element,^[^ ^2^ ^]^ hence its substitution with nonmagnetic ions will decrease the long‐range magnetic order and *T*_c_. It should be noted that variations in the lattice constant *a*_lat_ may also contribute to changes in $T_{c}^{*}$. We observe that the highest Al concentration studied here reduces *T*_c_ by about 55 K.

### 2.4 Influence of Al Doping on Magnetic Compensation Temperatures

Figure 3b shows the evolution of the magnetic compensation temperature *T*_M_ as a function of $P_{{\mathrm{Al}}_{2}O_{3}}$. Here, the changes are more drastic. *T*_M_ increases from about 190 K in undoped TbIG, consistent with our previous films,^[^ ^23^ ^]^ to above 360 K in the highest doped Al:TbIG. We note that the last data point (red diamond) corresponds to the ordering temperature of that film, since magnetic compensation was not observed until the disappearance of AHE. Our measurements clearly show that Al substitution, which is easily obtained by co‐sputtering, is an effective method to tune magnetic properties in typical garnet structures such as TbIG.

The overall behavior of magnetic compensation in TbIG—and in rare‐earth iron garnets (REIGs) more broadly—can be described by a mean‐field theory.^[^ ^2^ ^]^ Figure 3c presents a schematic illustration of the sublattice magnetizations of TbIG, plotted as |*M*_Fe_| and |*M*_Tb_|, where *M*_Fe_ and *M*_Tb_ denote the Fe (red) and Tb (blue) sublattice magnetizations, respectively. The Fe‐sublattice magnetization *M*_Fe_ arises from the uncompensated Fe^3 +^ moments located at the *a*(octahedral)‐sites and *d*(tetrahedral)‐sites, where 24 *d*‐site and 16 *a*‐site Fe^3 +^ moments are antiferromagnetically coupled within a garnet unit cell. The net magnetization is given by *M* = ||*M*_Fe_| − |*M*_Tb_||, considering that *M*_Fe_ and *M*_Tb_ are aligned antiparallel. In REIGs, the ferrimagnetic ordering is primarily governed by the Fe sublattice, and *M*_Fe_ exhibits temperature dependence similar to that of a ferromagnet [see the red curve in Figure 3c]. In contrast, the Tb^3 +^ moments are relatively weakly bound and exhibit paramagnetic behavior with a Curie–Weiss‐like temperature dependence, even in the magnetically ordered phase [see the blue curve in Figure 3c].^[^ ^2^, ^31^ ^]^ Nevertheless, the *M*_Tb_ sublattice magnetization remains finite (i.e., the Tb^3 +^ moments are polarized) even under zero external field due to exchange‐coupling fields (i.e., the mean field) arising from the ordered Fe^3 +^ sublattice. The magnetic compensation temperature *T*_M_ is defined as the temperature at which |*M*_Fe_| and |*M*_Tb_| intersect, i.e., where *M* = 0.

The increase in the magnetic compensation temperature *T*_M_ induced by Al substitution can be attributed to two primary factors as follows: the reduction of *M*_Fe_ due to Al substitution [see the red arrow labeled [A] in Figure 3c], and the enhancement of *M*_Tb_ resulting from the decrease in the lattice constant *a*_lat_ caused by Al substitution [blue arrow labeled [B]]. The former effect—the reduction in *M*_Fe_—can be explained by a decrease in the number of uncompensated *d*‐site Fe moments. The reduction in *M*_Fe_ induced by Al substitution has been well established in previous studies on Al‐substituted yttrium iron garnet (YIG) thin films.^[^ ^32^ ^]^ Although the site preference of Al in garnet structures remains a subject of debate,^[^ ^26^, ^28^, ^33^, ^34^ ^]^ our STEM analysis suggests a *d*‐site preference for Al substitution (see Section S6, Supporting Information), consistent with the observed increase in *T*_M_. It should also be noted that a reduction in the effective Curie temperature $T_{c}^{*}$ contributes to the decrease in *M*_Fe_, independent of the Al site preference. The latter effect—the enhancement of *M*_Tb_—can be understood as follows: the superexchange coupling, which governs the magnetic ordering in TbIG, is strengthened by the reduction of *a*_lat_, since the orbital overlap between Fe^3 +^/Tb^3 +^ and O^2 −^ ions increases as the interatomic distances decrease. As a result, the Tb^3 +^ moments experience a stronger effective mean field (i.e., exchange coupling field) from neighboring Fe^3 +^ moments, leading to an increase in *M*_Tb_. From the schematic illustration, it is evident that *T*_M_ shifts to higher temperatures as a consequence of the combined effects described above.

The above explanation for the enhancement of *T*_M_ due to the reduction in the lattice constant is supported by experimental observations. In our undoped thin‐film TbIG sample, the values of the magnetic compensation temperature and the lattice constant are *T*_M_ = 190 K and *a*_lat_ = 12.76 Å, respectively, whereas for bulk TbIG, they are *T*_M_ = 246 K^[^ ^14^ ^]^ and *a*_lat_ = 12.46 Å.^[^ ^22^ ^]^ It is commonly observed that thin‐film TbIG exhibits a larger lattice constant and a lower *T*_M_ compared to bulk samples; for example, TbIG grown on a yttrium scandium gallium garnet (YSGG) substrate shows *a*_lat_ = 12.58 Å and *T*_M_ = 215 K.^[^ ^35^ ^]^ Assuming a linear relationship between *T*_M_ and *a*_lat_, we estimate the variation in *T*_M_ arising solely from changes in the lattice constant, using the values for bulk and undoped thin‐film TbIG. The estimated values are indicated by the cross symbols connected by a dashed line in Figure 3b. These results indicate that the effect of lattice constant variation on *T*_M_ is substantial.

### 2.5 Observation of Intrinsic Exchange Bias in Al:TbIG

**Figure 4:**
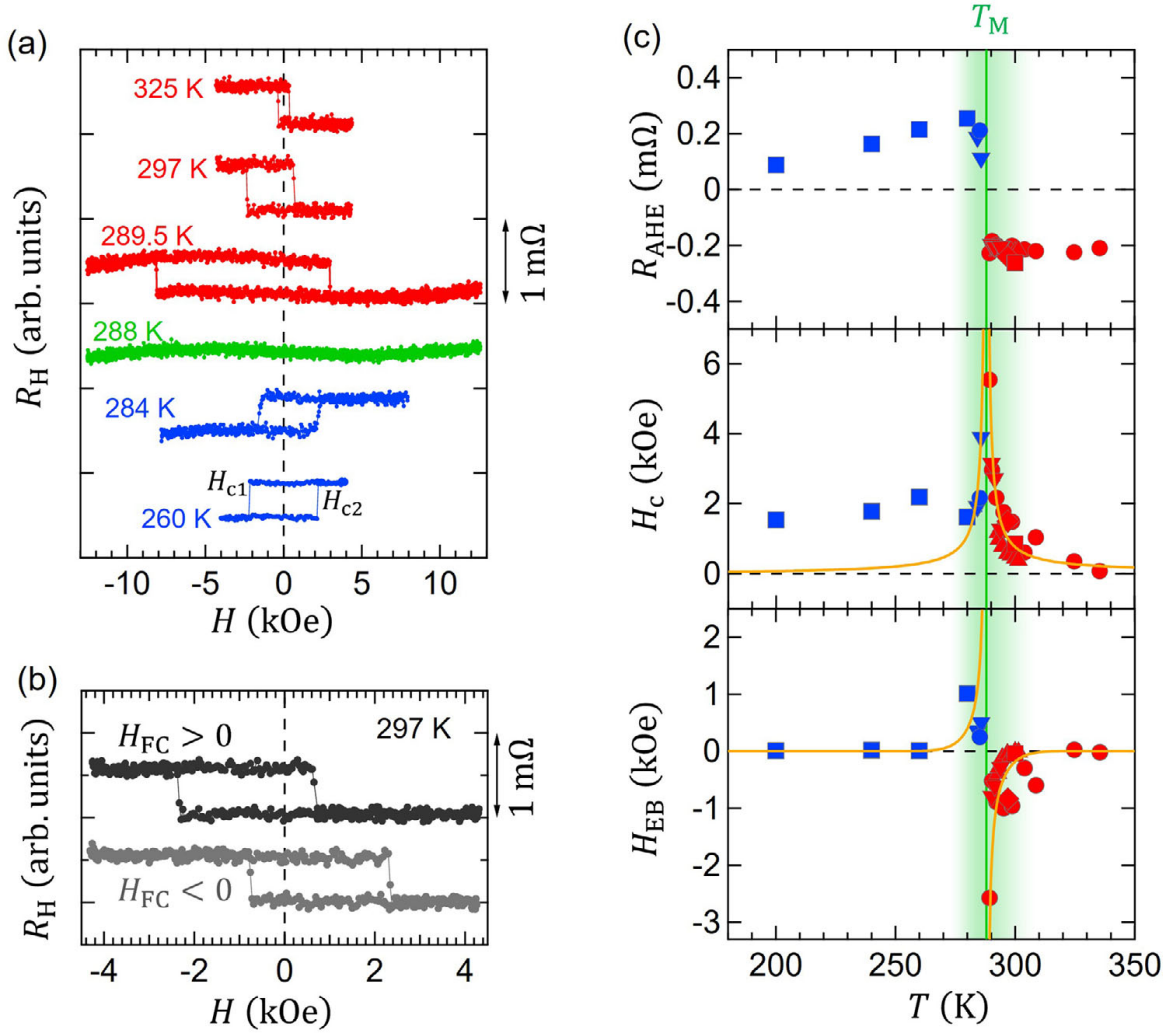
a) AHE hysteresis loops at selected temperatures showing the sign reversal of AHE and the emergence of exchange bias near TM b) Reversible control of exchange bias polarity via field cooling with ±13 kOe of applied field. Note that the AHE curves were obtained by subtracting the linear slopes of the ordinary Hall component from the measured Hall resistances. c) Plots of the AHE resistance *R*_AHE_ (upper panel), the coercivity *H*_c_ (middle panel), and the exchange bias field *H*_EB_ (lower panel). The vertical green line indicates the magnetic compensation temperature (*T*_M_ = 288 K). The yellow curves in the middle and lower panels are those derived from the mean‐field model (see the discussion section).

To examine the magnetization reversal anomalies near *T*_M_, we focus on Al:TbIG prepared with $P_{{\mathrm{Al}}_{2}O_{3}}$ = 65 W, where the concentration of Al atoms occupying Fe sites is estimated to be approximately 5% (see Section S1, Supporting Information). In this film *T*_M_ is 288 K, i.e., slightly below room temperature. **Figure** **4**a shows the AHE signals measured with an out‐of‐plane swept field for selected temperatures between 260  and 325 K. We find a significant exchange bias effect, more pronounced at 289.5 K, when temperature approaches *T*_M_. We find the exchange bias in other devices and films with different $P_{{\mathrm{Al}}_{2}O_{3}}$ (see Section S7, Supporting Information), confirming it is a general property of Al‐doped films in this study rather than a feature of this particular device.

Exchange bias can be modified by field cooling (FC).^[^ ^36^ ^]^ We attempt to control the sign of the exchange bias by performing a FC procedure, in which the sample is naturally cooled from 470 K ($>T_{c}^{*}$) to 297 K in ambient conditions in the presence of an out‐of‐plane bias field of ±13 kOe. The cooling proceeds at an average rate of 30 Kmin^−1^ from 470 to 320 K, followed by a 30‐min gradual cooling to the base temperature. Repeating the FC procedure for positive and negative fields leads to a reproducible change of the exchange bias sign as depicted in Figure 4b.

Figure 4c summarizes the temperature dependence of AHE resistance *R*_AHE_ (upper panel), coercivity *H*_c_ (middle panel), and the exchange bias field *H*_EB_ (lower panel). The values are calculated based on *H*_c_ = (*H*_*c*2_ − *H*_*c*1_)/2 and *H*_EB_ = (*H*_*c*1_ + *H*_*c*2_)/2, where *H*_c1_ (*H*_c2_) represents the magnetization reversal with the negative (positive) field [see 260 K data in Figure 4a]. The values of *R*_AHE_ were obtained from half of the jump at *H*_c2_ in the hysteresis loops. The behavior of *R*_AHE_ and *H*_c_ is consistent with typical magnetic compensation (see, e.g., Refs. [19, 23]), i.e., the sign change of *R*_AHE_ accompanied with a significant increase of *H*_c_ at *T*_M_. The magnitude of *H*_EB_ increases as the temperature approaches *T*_M_, and the sign of *H*_EB_ changes across *T*_M_. This behavior of *H*_EB_ near *T*_M_ is similar to that observed in other compensated ferrimagnetic systems (bulk materials).^[^ ^37^, ^38^, ^39^ ^]^ Data with different symbols represent measurements after different FC processes. Despite minor differences, after consecutive FC sequences, the magnetic behavior remains qualitatively the same.

**Figure 5:**
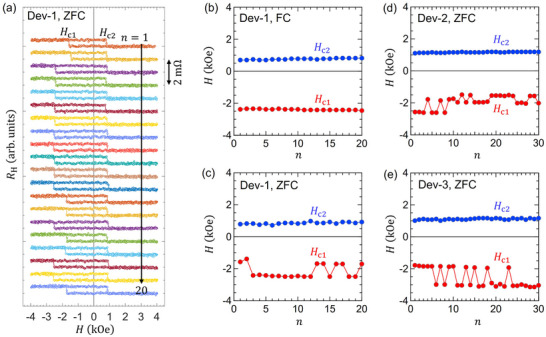
Hall effect measurement results after zero‐field cooling (ZFC): a) Twenty AHE hysteresis loops measured consecutively after ZFC. b,c) Plots of the switching fields *H*_c1_ and *H*_c2_ as a function of the cycle number *n* in the case of b) field cooling and c) zero‐field cooling. Note that the data in (a)‐(c) were obtained from the same device. d,e) Plots of *H*_c1_ and *H*_c2_ for different devices on the same sample.

The exchange bias effect becomes stochastic in the case of zero‐field cooling (ZFC), i.e., the same temperature process but no applied field. **Figure** **5**a displays twenty AHE hysteresis loops recorded sequentially at 297 K after ZFC. Interestingly, the switching field *H*_c1_ randomly changes during consecutive cycles, while *H*_c2_ remains constant. We plot *H*_c1_ and *H*_c2_ as a function of the cycle number (*n*) for FC in Figure 5b and for ZFC in Figure 5c measured on the same device (coined Dev‐1). In the case of FC, the exchange bias is strictly deterministic within a narrow switching field window, but with ZFC, *H*_c1_ tends to fluctuate between two distinct values with no sign of training effects typically observed in antiferromagnet/ferromagnet bilayers.^[^ ^36^ ^]^ Examination of other devices (Dev‐2 and Dev‐3) shows qualitatively the same behavior as displayed in Figure 5d,e (see Section S8, Supporting Information for more data on different devices). Interestingly, the value of *H*_c1_ tends to stochastically alternate between two or three values, depending on the device under test, in all cases. There is a reason why all the data in Figure 5c–e show a negative exchange bias. This is because all the measurements start from a large positive field of *H* ≈ +4 kOe, which sets the exchange bias direction prior to the field sweep. When reversing the initial field, i.e., starting the sweep from *H* ≈ −4 kOe, the exchange bias reverses sign (see Section S9, Supporting Information). The collective data in Figures 4 and 5 demonstrate that single Al:TbIG layers exhibit a large and intrinsic exchange bias effect whose sign and stochasticity can be controlled by moderate external fields and field cooling processes.

## 3 Discussion

### 3.1 Origin of the Intrinsic Exchange Bias: *T*_M_‐Distribution Model

We discuss the origin of the exchange bias observed near *T*_M_. The overall characteristics of the exchange bias in the present system are similar to those observed in other bulk compensated ferrimagnets like orthoferrites,^[^ ^37^, ^40^ ^]^ suggesting that the intrinsic exchange bias in the present case also originates from a bulk mechanism. It was proposed that the exchange bias may appear near *T*_M_ by considering two possible domain states.^[^ ^41^ ^]^ However, our extensive experimental data point to a more complex scenario beyond domain energy arguments. We thus propose an alternative and improved phenomenological model, discussed below, for the origin of the exchange bias by considering the presence of disorder in the network of magnetic Fe^3 +^ caused by partial Al^3 +^ substitution.

**Figure 6:**
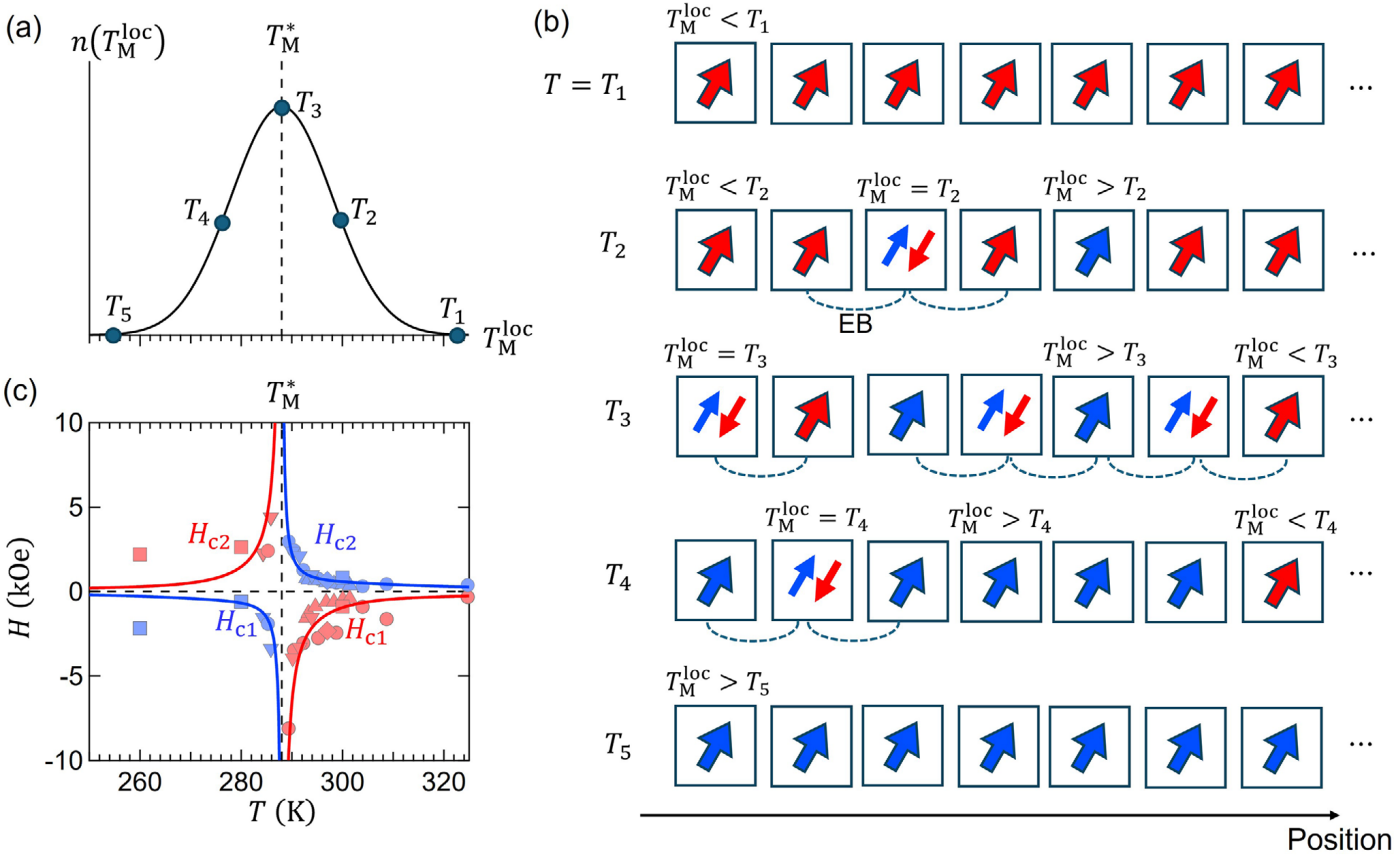
a) A Gaussian distribution profile [$n(T_{M}^{\mathrm{loc}})$] of the local compensation temperature ($T_{M}^{\mathrm{loc}}$). b) Schematic illustration of possible local magnetic states at different temperatures *T*_*k*_ (*k* = 1, ⋅⋅⋅, 5) indicated in (a). The red (blue) thick arrow represents a local FM state dominated by Fe^3 +^ (Tb^3 +^) magnetic moments. The pair of red and blue arrows indicates a local AFM state realized at $T_{M}^{\mathrm{loc}}$. c) Temperature dependence of the switching fields *H*_c1_ and *H*_c2_ (solid lines) calculated using Equation (2) with the Gaussian distribution of *U*(*T*) [Equation (1)]. The experimental data of *H*_c1_ and *H*_c2_ (which correspond to *H*_c_ and *H*_EB_ in Figure 4c are plotted together. The red curves represent the switching fields *H*_c1_ for $T>T_{M}^{*}$ and *H*_c2_ for $T<T_{M}^{*}$, while the blue curves correspond to *H*_c2_ for $T>T_{M}^{*}$ and *H*_c1_ for $T<T_{M}^{*}$.

We propose a Gaussian distribution of *T*_M_ in the film due to local fluctuations of Al^3 +^ concentration. **Figure** **6**a shows the distribution function [$n(T_{M}^{\mathrm{loc}})$] of the local compensation temperature ($T_{M}^{\mathrm{loc}}$) in the film. In this model, local compensation temperatures are represented as local domains that can interact with nearby domains, as illustrated in Figure 6b. Here, ‘domain’ refers to a local region with a specific $T_{M}^{\mathrm{loc}}$ due to spatial variations in Al content, and should not be confused with conventional magnetic domains. Here, each row corresponds to a different global temperature *T*_*k*_ (*k* = 1, ⋅⋅⋅, 5). Then at each temperature, each domain takes one of the following three possible states: *i)* Fe‐dominated FM state (for $T_{M}^{\mathrm{loc}}<T_{k}$); *ii)* Tb‐dominated FM state (for $T_{M}^{\mathrm{loc}}>T_{k}$); and *iii)* compensated AFM state (for $T_{M}^{\mathrm{loc}}=T_{k}$). When the neighboring domains have a combination of *i)* and *iii)* [or *ii)* and *iii)*], the FM domain acquires an exchange bias from the neighboring AFM domain. Therefore, exchange bias is expected to occur throughout the entire temperature range of the distribution of $T_{M}^{\mathrm{loc}}$ but with different global behavior. In this distribution model, we assume the unidirectional exchange bias anisotropy energy *U*(*T*) has the Gaussian form (see Section S10, Supporting Information for the derivation):

(1) $$U\left(T\right)\approx U_{0}\exp\left[-\frac{{\left(T-T_{M}^{*}\right)}^{2}}{2\sigma^{2}}\right]$$

where *U*_0_ is a coefficient giving the magnitude of *U*(*T*), $T_{M}^{*}$ is the mean value of the distribution, σ is the standard deviation. This model is expected to be valid when the characteristic domain size is sufficiently small to satisfy the statistical large‐number condition. We consider a nanometer‐scale domain size, corresponding to approximately 10–1000 unit cells per domain. In the present case, the Hall‐bar cross volume is 7.5 µm × 3 µm × 25 nm, and to accommodate a large number of domains (e.g., 1 × 10^6^), each domain would need to be approximately 8 × 8 × 8 nm^3^ in size. We also note that a Gaussian distribution (of elemental concentration) has previously been employed in a different context to account for the complex magnetic behavior of CoGd ferrimagnetic alloys.^[^ ^42^ ^]^

By employing the above distribution function, the temperature dependence of the switching field (*H*_sw_) can be described within the framework of the mean‐field theory as follows:^[^ ^37^ ^]^

(2) $$H_{\mathrm{sw}}=-\left(\frac{\Delta E_{z}^{\mathrm{sw}}}{2M_{\mathrm{Fe}}}\right)\left(\frac{T-\theta_{w}}{T-T_{M}^{*}}\right)$$

where $\Delta E_{z}^{\mathrm{sw}}$ is the Zeeman energy at switching, *M*_Fe_ is the sublattice magnetization of the Fe^3 +^ ions and θ_w_ is the Weiss temperature associated with the interactions between RE^3 +^ moments. Note that *T*_M_ is replaced with $T_{M}^{*}$ in the present case. By including the uniaxial anisotropy (*K*) and unidirectional exchange bias anisotropy (*U*), the Zeeman energy at switching can be expressed as $\Delta E_{z}^{\mathrm{sw}}=K\pm U$. The positive sign (*K* + *U*) corresponds to hard switching due to the exchange bias, while the negative sign (*K* − *U*) corresponds to easy switching. For the Weiss temperature θ_w_, we adopt the value of Tb_3_Ga_5_O_12_ (θ_w_ = −7 K).^[^ ^43^ ^]^

Figure 6c shows the calculated *H*_c1_ and *H*_c2_ using the above model. The experimental data of *H*_c1_ and *H*_c2_ (corresponding to the data in Figure 4c) are also plotted together for comparison. We find an excellent agreement between the predicted switching fields and the experimental data, except for low temperatures where the magnetic anisotropy tends to increase contrary to our constant anisotropy assumption. Likewise, the values of *H*_c_ and *H*_EB_ derived from the model are also plotted in Figure 4c (yellow curves), respectively, again, showing an excellent match. The input parameters are determined as follows. From the observed sign‐reversal behavior of AHE, we set $T_{M}^{*}=288$ K. The values of σ = 10 K, *K*/(2|*M*_Fe_|) = 30 Oe and *U*_0_/(2|*M*_Fe_|) = 15 Oe are empirically determined so that the model fit passes through the center of the scattered data points (*H*_c1_ for $T>T_{M}^{*}$). Therefore, in the present case, $\Delta E_{z}^{\mathrm{sw}}/\left(2M_{\mathrm{Fe}}\right)=45$ Oe (for *M*_Fe_ > 0) corresponds to the hard switching field, while $\Delta E_{z}^{\mathrm{sw}}/\left(2M_{\mathrm{Fe}}\right)=-15$ Oe (for *M*_Fe_ < 0) corresponds to the easy switching field. Note that in Equation (2), the overall sign reversal of the switching fields (and the unidirectional anisotropy) across $T_{M}^{*}$ arises from the sign change of the denominator, $\left(T-T_{M}^{*}\right)$. In addition, the validity of the Gaussian distribution of *U*(*T*) was checked by a further detailed analysis (see Section S11, Supporting Information), in which different types of distribution functions were also tested. Among them, the Gaussian function provided the best fit to the experimental data.

The present model works remarkably well with our data and it can be applied to any compensated ferrimagnetic compounds with disorder in the magnetic sites. The width of the distribution (∝σ) reflects the degree of disorder. Exchange bias is not expected in a fully ordered system corresponding to the limit σ → 0, where the Gaussian becomes delta‐function‐like. Previous studies reported exchange bias in substituted REIGs near *T*_M_, where the origin was attributed to inhomogeneities localized at the surface of the film.^[^ ^44^ ^]^ Our films are relatively thick and the contribution from the surface states can be neglected. We stress that the exchange bias was observed regardless of two different surface conditions (i.e., the presence or absence of the TbIG(2 nm) layer) ruling out the surface arguments for the origin of exchange bias (see Section S2, Supporting Information). Since real materials inherently contain defects that potentially introduce disorder in the system, the present model suggests a possibility of exchange bias near *T*_M_ in any given ferrimagnets provided that structural defects exist (see Section S7, Supporting Information).

### 3.2 Stochastic Exchange Bias: Phenomenological Description Based on Free Energy Landscape

We discuss the intriguing stochasticity of the exchange bias effect observed after the ZFC process. An additional observation to be noted is that a similar unidirectional stochasticity was detected in the *H*_c1_ values between different FC processes (within the same FC, *H*_c1_ was constant and deterministic), even when following the same FC protocol, as shown in Figure 6c. This suggests that the exchange bias state, particularly the value of *H*_c1_, is stochastically determined during the cooling process.

**Figure 7:**
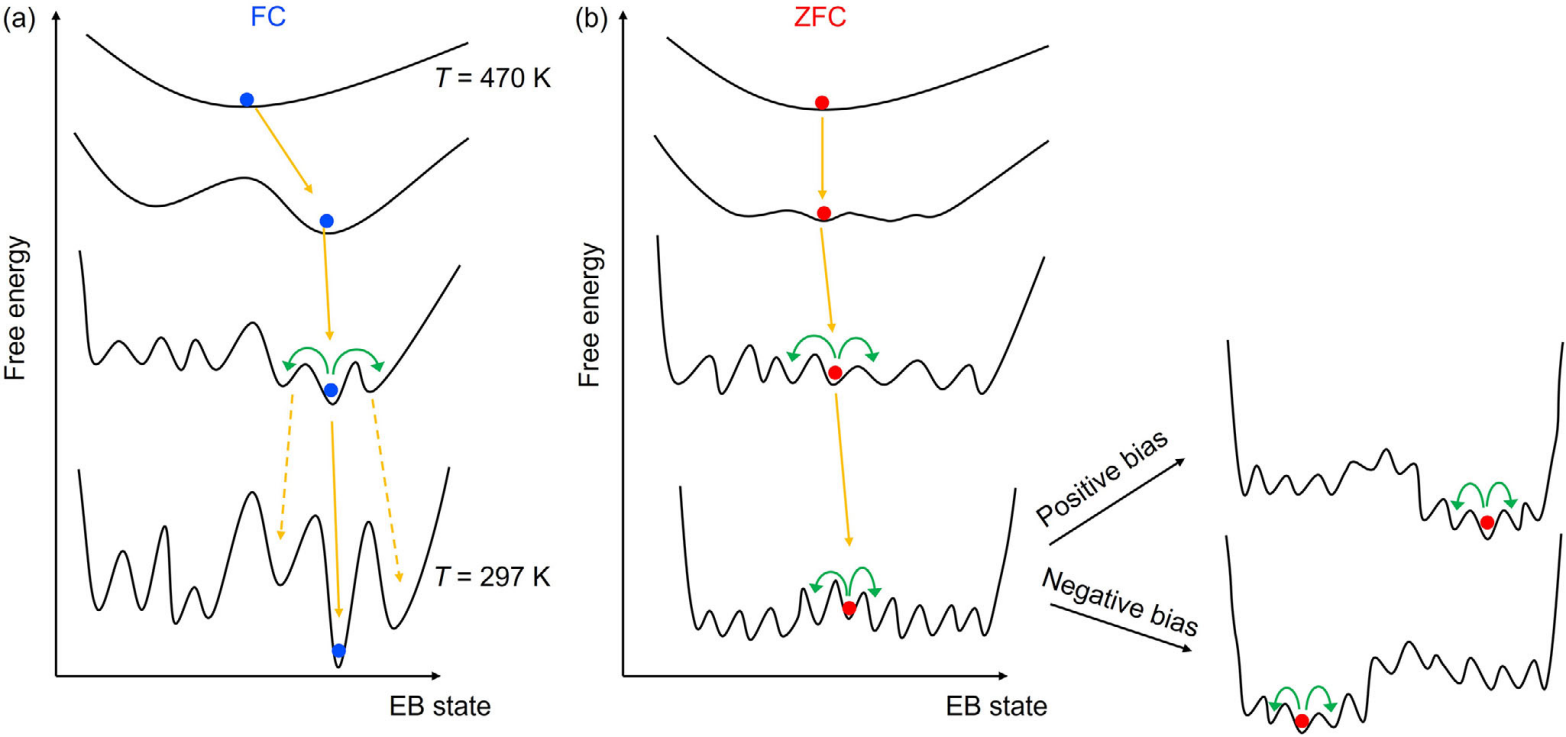
Illustration of a possible evolution of the free energy landscape and the exchange bias state during a) FC and b) ZFC cooling to room temperature. Yellow arrows indicate the transition of exchange bias states during the cooling process, while green arrows show that the system can hop between different energy valleys. In the ZFC case, the energy landscape can be further modified by the initial application of a magnetic field, depending on its direction (i.e., positive bias or negative bias).

In the following, we propose a possible explanation for the stochasticity based on a multi‐valley free energy landscape for the present ferrimagnetic system, where randomness and frustration are expected to arise due to the partial substitution of Fe^3+^ with Al^3+^. This idea is inspired by the analogy to spin glasses, in which such a multi‐valley free energy structure is expected in the mean‐field theory.^[^ ^45^ ^]^ **Figure** **7**a illustrates how the free energy landscape and the exchange bias state evolve during an FC process. An external magnetic field introduces an asymmetry into the free energy landscape, making certain valleys deeper. As a result, the system is more likely to settle into one of these deeper valleys. At higher temperatures, due to the presence of relatively small energy barriers, the system can still explore multiple valleys and may become trapped in one of them (represented by the dashed yellow arrows)—accounting for the observed variation in *H*_c1_ values. In the case of ZFC [see Figure 7b], where no bias is applied during cooling, many shallow valleys of comparable depth exist, and the system randomly falls into one of them. The initial application of an external magnetic field at room (measurement) temperature further modifies the energy landscape and the exchange bias state, depending on the field direction (i.e., positive bias or negative bias). Due to the small energy barriers associated with these metastable states, the system may transition between different valleys during field sweep measurements as a result of thermal fluctuations and/or the applied external field. The unidirectional nature of the stochastic behavior might be explained by the asymmetric structure of the switching‐field curves (see Section S12, Supporting Information), although this remains to be clarified.

### 3.3 A Possible Role of the TbIG Capping

Finally, we discuss a possible role of the 2‐nm TbIG capping layer. In our measurements on the uncapped sample (in which Pt was deposited directly onto Al:TbIG without a 2‐nm‐TbIG capping), the intrinsic exchange bias was clearly observed, including by a sign reversal across *T*_M_ and the ability to control the exchange‐bias direction via an external magnetic field (*H*_FC_) applied during FC, as well as by the initial bias direction in ZFC protocols (see Section 2, Supporting Information). Note that the lattice constant of the uncapped sample is nearly identical to that of the main capped sample. However, the uncapped sample did not exhibit clear deterministic behavior and instead showed stochastic behavior regardless of whether field cooling (FC) or zero‐field cooling (ZFC) protocols were applied (see Section S2, Supporting Information). In other words, the deterministic nature of the exchange bias in the uncapped sample was unstable. These results suggest that the 2‐nm TbIG capping layer plays a role in stabilizing the deterministic exchange bias state. This interpretation is supported by the fact that the capped region is expected to have a lower Al concentration due to an Al concentration gradient within the interfacial 2‐nm TbIG capping layer (see Section S5, Supporting Information). As a result of this gradient, the local *T*_M_ at the interface is expected to be lower than that in the bulk, leading to a locally uniform Fe‐dominant state that serves as a stabilizing factor for deterministic exchange bias under FC. Therefore, the capping layer—specifically, the presence of an Al concentration gradient—may be one of the key factors enabling control over the switching between deterministic and stochastic states via FC/ZFC protocols. Additionally, we note that the magnetic state is sensitive to Al doping and/or the value of *T*_M_. For example, in samples with higher Al levels (e.g., $P_{{\mathrm{Al}}_{2}O_{3}}=87$ W), we observed qualitatively different magnetic behavior such as a triple‐hysteresis loop, which will be reported in a separate publication. The observed difference in compensation temperatures (*T*_M_ = 288 K for the capped sample and *T*_M_ = 315 K for the uncapped sample) also points to intrinsic differences that may influence stochastic/deterministic behavior. Further studies are required to clarify the detailed conditions that govern this controllability.

## 4 Conclusion

We have demonstrated a controllable and scalable strategy to tune the magnetic compensation temperature of TbIG by substituting Fe with Al, achieving *T*_M_ values above room temperature. This chemical tuning also induces a giant, intrinsic exchange bias effect—unprecedented in garnet systems—whose sign and stochasticity can be modulated via external magnetic fields and thermal history. The phenomenological model based on a Gaussian distribution of local *T*_M_ values captures the essential features of the observed behavior, highlighting the role of disorder in enabling exchange bias in compensated ferrimagnets. These results open new avenues for engineering garnet‐based spintronic devices with tunable magnetic functionalities at ambient conditions.

## 5 Experimental Section

### Sputtering

The co‐sputtered TbIG‐Al_2_O_3_ layer (nominal thickness: 23 nm) and a 2‐nm TbIG capping layer were deposited on a GGG(111) substrate at 800 °C using RF sputtering using stoichiometric targets. Prior to sputtering, the GGG(111) substrate underwent plasma cleaning (performed with 30 W excitation power under 3 mTorr Ar gas at room temperature). The co‐sputtered layer's nominal thickness of 23 nm was determined by the deposition rate of single‐layer TbIG sputtering, with a fixed sputtering time of approximately 1 h. The sputtering power for TbIG was set at 150 W, while the power for Al_2_O_3_ ($P_{{\mathrm{Al}}_{2}O_{3}}$) was varied. The deposition was carried out at a working pressure of 3 mTorr using a gas mixture of Ar and O_2_ in a 30:2 ratio.^[^ ^23^ ^]^ A 4‐nm Pt layer was subsequently deposited on the oxide layer via DC magnetron sputtering without breaking vacuum, using a working pressure of 3 mTorr and a power of 50 W at room temperature.

### Sample Characterization

X‐ray diffraction measurements were carried out with an in‐house diffractometer equipped with a monochromator (Cu‐Kα1). The microstructure of the sample was analyzed using scanning transmission electron microscopy (STEM) at the Joint Electron Microscopy Center at ALBA (Cerdanyola del Vallès, Spain). A double‐corrected ThermoFisher Spectra 300 (S)TEM microscope operated at 300 kV, equipped with a monochromator and a continuum spectrometer with a K3 direct electron detection camera, was used. High‐angle annular dark field (HAADF) images were captured with a semiconvergence angle of 19.5 mrad. Electron energy loss spectroscopy (EELS) spectra were acquired with a collection angle of approximately 21.4 mrad, a probe current of about 100 pA, and a dwell time of 2 ms. To reduce random noise in the EELS spectrum images, a principal component analysis filter was applied after acquisition.

### Device Fabrication and Hall Effect Measurement

Hall‐bar devices [Figure 1b] used in this study were fabricated via photolithography and inductively coupled plasma reactive ion etching. Transport measurements were primarily performed using an AC lock‐in harmonic technique with an excitation amplitude of 1 V at a frequency of 1092 Hz, utilizing a lock‐in amplifier (Zurich Instruments Ltd.). Temperature‐dependent measurements above 270 K were conducted using custom‐built heating and cooling setups under atmospheric conditions. For temperatures below 270 K, data were collected using a commercial physical property measurement system (Quantum Design).

## Conflict of Interest

The authors declare no conflict of interest.

## Supporting information

Supporting Information

## Data Availability

The data that support the findings of this study are available from the corresponding author upon reasonable request.
