# Supplementary material for: Tunable Magnetism and Intrinsic Exchange Bias in Al‐Substituted Terbium Iron Garnet
Source: Adv Mater. 2025 Sep 18;38(2):e10669. doi: 10.1002/adma.202510669 (PMC12783968; doi:10.1002/adma.202510669)
Supplement: Supplementary file 1 — Supporting Information [file ADMA-38-e10669-s001.pdf]

ADVANCED MATERIALS

Supporting Information

for *Adv. Mater.*, DOI 10.1002/adma.202510669

Tunable Magnetism and Intrinsic Exchange Bias in Al-Substituted Terbium Iron Garnet

Takayuki Shiino, Matteo Fettizio, Saúl Estandía and Can Onur Avci**

Supporting Information for Tunable Magnetism and Intrinsic Exchange Bias in Al-Substituted Terbium Iron Garnet

Takayuki Shiino,[1,*] Matteo Fettizio,[1] Saúl Estandía,[1] and Can Onur Avci[1,†]

[1] *Institut de Ciència de Materials de Barcelona (ICMAB-CSIC), Campus de la UAB, Bellaterra, 08193, Spain*
(Dated: September 16, 2025)

CONTENTS

S1. Estimated concentration of aluminum atoms in the co-sputtered layer 2
S2. AHE for a system without a TbIG(2nm) capping layer 3
S3. In-plane-anisotropy-like behavior in $P_{\mathrm{Al_2O_3}} = 109$ W 5
S4. XRD analysis for aluminum oxide impurity phase 6
S5. Supplementary note for the STEM data 7
Note for Figure 2(c) 7
EELS intensity profiles 7
Quantifying relative atomic composition of Fe and Al by EELS 7
S6. d-site preference of Al substitution 9
S7. Exchange bias in $P_{\mathrm{Al_2O_3}} = 0$ and 43 W 10
S8. Examination of ZFC stochastic exchange bias in different devices 11
S9. ZFC stochastic exchange bias for the initial negative bias case 12
S10. Derivation of the temperature dependence of exchange bias anisotropy 13
S11. Validity of Gaussian distribution 15
S12. Possible explanation for the unidirectional nature of the stochastic EB 17

References 18

S1. Estimated concentration of aluminum atoms in the co-sputtered layer

We estimated the concentration of Al atoms in the Fe sites of Al:TbIG as follows. There are 40 Fe atoms in the cubic unit cell of TbIG. For simplicity, we assume the bulk value of the lattice constant of TbIG ($a_{\rm TbIG} = 12.436$ Å) [1]. The volume of the unit cell of TbIG is $V_{\rm uc}^{\rm TbIG} = a_{\rm TbIG}^3$. The thickness of TbIG (only) on GGG(111) substrate is $t_{\rm TbIG} = 23$ nm for the present sputtering condition. Then, the number of Fe atoms ($N_{\rm Fe}$) in the film is given as follows:

$$N_{\rm Fe} = 40 \times \frac{t_{\rm TbIG} A_{\rm sub}}{V_{\rm uc}^{\rm TbIG}}, \tag{1}$$

where $A_{\rm sub}$ ($= 5 \times 5$ mm^2) is the surface area of the substrate. In the case of Al_2O_3, the unit cell is not cubic but trigonal. There are 4 Al atoms in the unit cell of Al_2O_3. The volume of the unit cell of Al_2O_3 is $V_{\rm uc}^{\rm Al_2O_3} = \frac{3\sqrt{3}}{2} a_{\rm Al_2O_3}^2 c_{\rm Al_2O_3}$ where $a_{\rm Al_2O_3} = 4.76$ Å and $c_{\rm Al_2O_3} = 12.99$ Å [2]. For simplicity, we approximate the unit cell of Al_2O_3 as an equivalent cube, and the number of Al atoms ($N_{\rm Al}$) in Al_2O_3 deposited on GGG(111) substrate should be given as follows:

$$N_{\rm Al} = 4 \times \frac{t_{\rm Al_2O_3} A_{\rm sub}}{V_{\rm uc}^{\rm Al_2O_3}}, \tag{2}$$

where $t_{\rm Al_2O_3}$ is the thickness of Al_2O_3 (only) on GGG(111) substrate. In our sputtering condition and system, $t_{\rm Al_2O_3}[{\rm nm}] = 0.0737[{\rm nm/W}] \times P_{\rm Al_2O_3}[{\rm W}]$. The concentration of Al atoms in the Fe sites of TbIG can be estimated by

$$\frac{N_{\rm Al}}{N_{\rm Fe} + N_{\rm Al}} = \frac{4(t_{\rm Al_2O_3}/V_{\rm uc}^{\rm Al_2O_3})}{40(t_{\rm TbIG}/V_{\rm uc}^{\rm TbIG}) + 4(t_{\rm Al_2O_3}/V_{\rm uc}^{\rm Al_2O_3})}. \tag{3}$$

Figure S1 presents the $P_{\rm Al_2O_3}$ dependence of the estimated concentration of Al atoms in the Fe sites of TbIG in the co-sputtered layer. Here all Al atoms (from Al_2O_3) are assumed to be in the Fe sites.

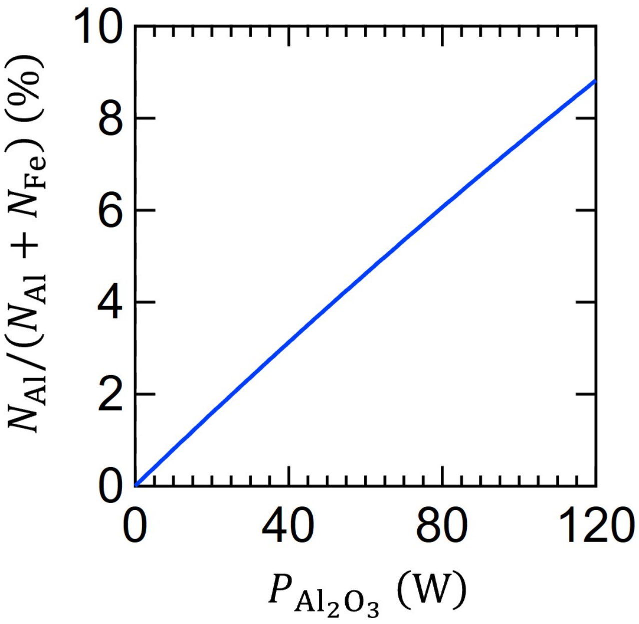

FIG. S1. Estimated concentration of Al atoms in Al:TbIG in our sputtering system and conditions.

S2. AHE for a system without a TbIG(2nm) capping layer

The primary samples studied in this paper include a TbIG (2 nm) capping layer deposited on top of the cosputtered layer. To examine the role of the capping layer, particularly regarding exchange bias, we investigated a system without the TbIG (2 nm) capping layer for $P_{\mathrm{Al_2O_3}} = 65$ W (see Fig. S2(a)). Figure S2(b) illustrates the field dependence of the AHE resistance at various temperatures. Figures S2(c)-(e) present the temperature dependence of the amplitude of the AHE resistance (R_{AHE}), the coercivity (H_{c}), and the exchange bias field (H_{EB}), respectively. The magnetic compensation temperature was determined to be approximately $T_{\mathrm{M}} = 315$ K. The overall behavior is qualitatively similar to that of the primary sample with the TbIG (2 nm) capping layer, although T_{M} is slightly higher in this case. An exchange bias effect is clearly observed near T_{M}, even without the TbIG (2 nm) capping layer. This result indicates that the interfacial condition between the co-sputtered layer and the Pt layer is independent of the origin of EB in the present system.

We examined the stochastic and deterministic exchange bias behavior in the uncapped sample. Figures S3(a)–(f) show the switching fields H_{c1} and H_{c2} as a function of cycle number n, obtained from anomalous Hall effect (AHE) measurements on the uncapped sample. The direction (sign) of the exchange bias can be controlled either by the external magnetic field H_{FC} applied during field cooling (FC) [Figs.S3(a) and (b)], or by the initial bias direction (i.e., the sign of the initial field used for the first field sweep) in zero-field cooling (ZFC) conditions [Figs.S3(c) and (d)]. These features are consistent with those observed in the primary sample capped with 2-nm TbIG. Note that the data in panels (a)–(d) were obtained from the uncapped device labeled No-cap-Dev-1, whereas panels (e) and (f) present additional measurements from a second device (No-cap-Dev-2) under FC ($H_{\mathrm{FC}} > 0$) and ZFC (positive bias) conditions. Interestingly, in contrast to the main capped sample, the uncapped sample does not exhibit a clearly deterministic exchange bias behavior after FC [see Figs.S3(a), (b), and (e)]. In other words, the deterministic state is unstable, and a stochastic exchange bias state appears to exist inherently in the uncapped sample. It is worth noting that the data in Fig.S3(d) do not show visible stochasticity; however, this likely reflects a pseudo-deterministic behavior that emerges from an underlying stochastic state. These results suggest that the 2-nm TbIG capping layer plays a crucial role in stabilizing the deterministic exchange bias state and enabling robust control over the switching between deterministic and stochastic states via FC and ZFC protocols.

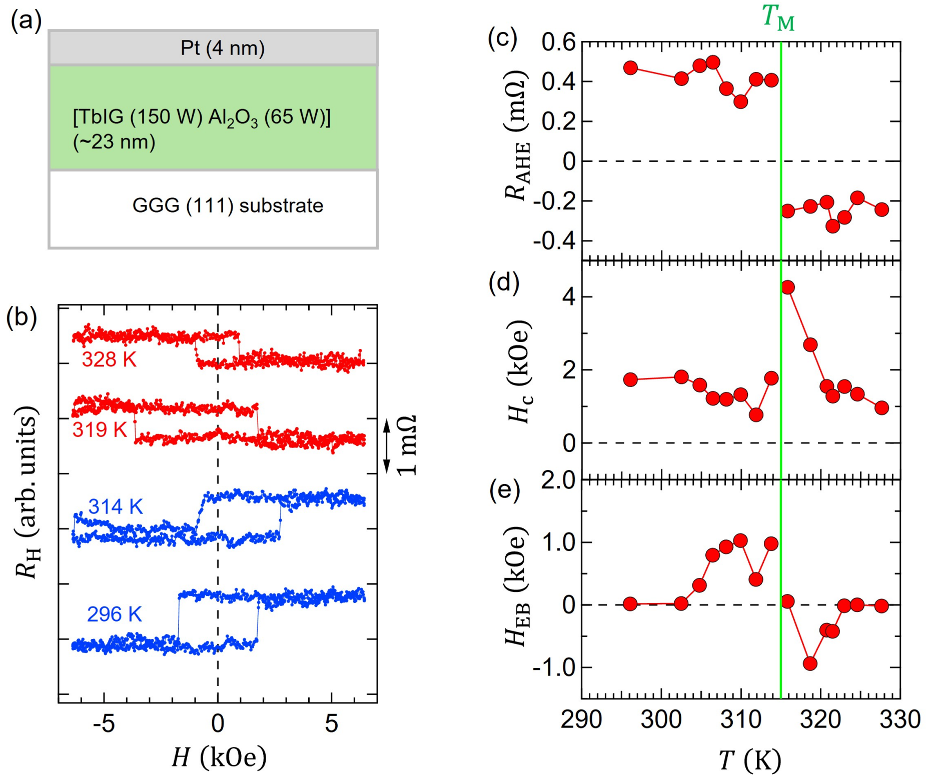

FIG. S2. (a) Schematic illustration for the sample stack without a TbIG(2 nm) capping layer. (b) Magnetic field dependence of the Hall resistance. Note that linear contributions from the ordinary Hall effect are subtracted. (c-e), Temperature dependence of (c) the magnitude of AHE (R_{AHE}), (d) coercivity (H_{c}) and (e) exchange bias field (H_{EB}).

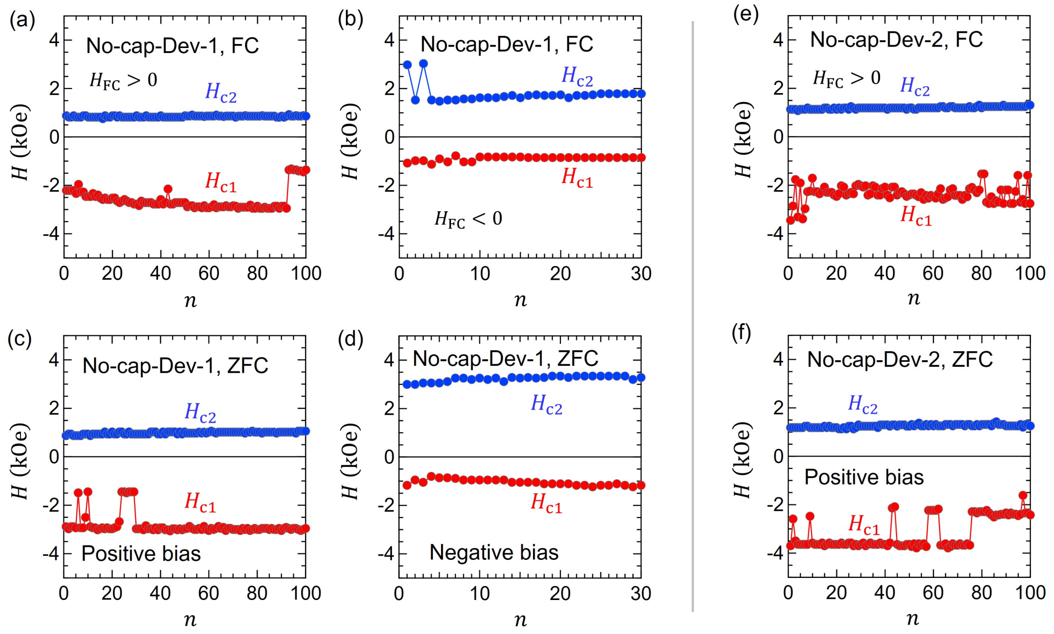

FIG. S3. Hall effect measurement results of the uncapped sample after field cooling (FC) and zero-field cooling (ZFC). Switching fields H_{c1} and H_{c2} are plotted as a function of cycle number n for (a) FC with a positive field ($H_{\mathrm{FC}} = +13$ kOe), (b) FC with a negative field ($H_{\mathrm{FC}} = -13$ kOe), (c) ZFC with an initial positive bias field, and (d) ZFC with an initial negative bias field. The data in panels (a)–(d) were obtained from the device labeled No-cap-Dev-1. Panels (e) and (f) show the corresponding measurements for the same FC and ZFC conditions as in (a) and (c), respectively, using a different device labeled No-cap-Dev-2. All measurements were performed at $T = 316.6 \pm 0.2$ K.

S3. In-plane-anisotropy-like behavior in $P_{\mathrm{Al_2O_3}} = 109$ W

Figure S4 shows the Hall resistance R_{H} for $P_{\mathrm{Al_2O_3}} = 109$ W measured at several temperatures from 298 K to 363 K. The AHE at room temperature appears to saturate above 2 kOe, and the overall behavior suggests that the total anisotropy of the present system for $P_{\mathrm{Al_2O_3}} = 109$ W is an in-plane anisotropy. The peculiar behavior observed in the low-field region ($|H| < 2$ kOe) is attributed to the in-plane component of spin Hall magnetoresistance. The positive sign of the AHE indicates that the Tb moments predominantly contribute to the total magnetization, while the Fe moments are aligned opposite to the direction of the applied field.

At slightly higher temperatures (around 320 K), the system exhibits PMA behavior. This can be explained as follows. The crystallographic anisotropy would still be PMA at room temperature, but the magnitude of PMA for $P_{\mathrm{Al_2O_3}} = 109$ W is smaller than that for lower $P_{\mathrm{Al_2O_3}}$ values. Therefore, because of the demagnetization effect, the total anisotropy becomes in-plane (easy-plane) anisotropic. However, at slightly higher temperatures (closer to the Curie temperature $T_{\mathrm{c}}^{*} = 363$ K), the magnetization (thus demagnetization energy) becomes smaller and the crystallographic PMA overcomes the demagnetization energy. As the system temperature approaches $T_{\mathrm{c}}^{*} = 363$ K, again, an in-plane-anisotropy behavior appears. This would be attributed to the change of (crystallographic) anisotropy and/or thermal spin fluctuation enhanced near the critical temperature $T_{\mathrm{c}}^{*} = 363$ K.

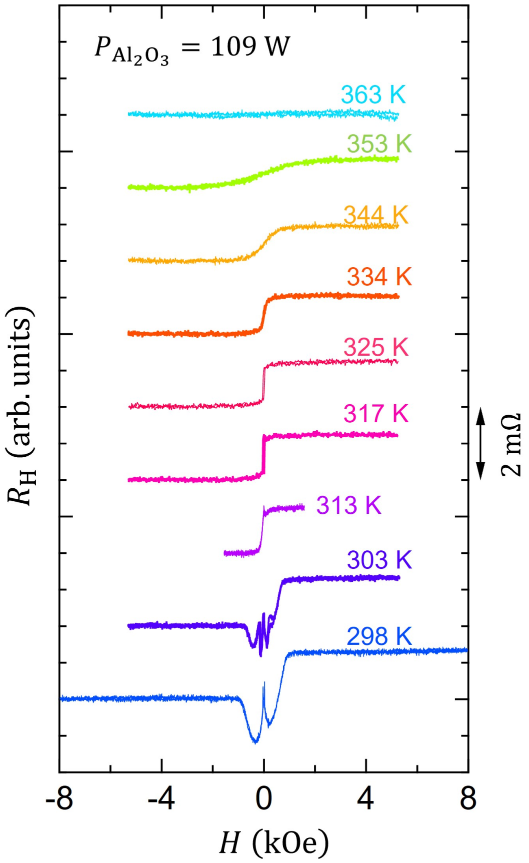

FIG. S4. R_{H} vs H curves for $P_{\mathrm{Al_2O_3}} = 109$ W near at various temperatures above room temperature. A linear contribution from the ordinary Hall effect has been subtracted in the plots.

S4. XRD analysis for aluminum oxide impurity phase

Figure S5 shows the x-ray diffraction (XRD) patterns of Al_2O_3 deposited on GGG(111) [Al_2O_3/GGG(111)] and the co-sputtered system with $P_{Al_2O_3} = 0$, 43, 65, 87, and 109 W. The XRD measurement result for a GGG(111) substrate is also included as a reference. The peaks observed in the Al_2O_3/GGG(111) and the co-sputtered system of $P_{Al_2O_3} = 109$ W around $2\theta = 24.9$ degrees are attributed to the Bragg reflection of α-Al_2O_3(012) [3]. For the low Al-content samples ($P_{Al_2O_3} < 100$ W), clear peaks were not observed.

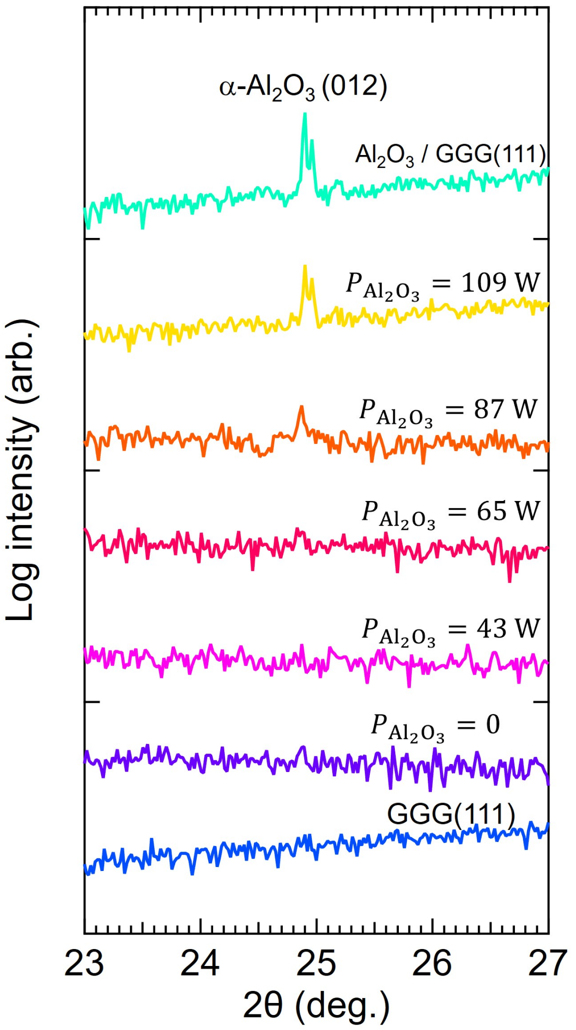

FIG. S5. XRD patterns of Al_2O_3/GGG(111), the co-sputtered systems for $P_{Al_2O_3} = 0$, 43, 65, 87 and 109 W and a GGG(111) substrate. Note that the double peak structure observed in Al_2O_3/GGG(111) and $P_{Al_2O_3} = 109$ W are attributed to the presence of both Cu-Kα1 and Kα2 peaks.

S5. Supplementary note for the STEM data

Note for Figure 2(c)

Figure 2(c) in the main text has been geometrically corrected to account for drift effects. Figure S6(a) shows the raw HAADF image viewed along the [1-10] direction. This raw image is distorted due to drift effects. To correct for this distortion, we adjusted the HAADF image by referencing the known lattice structure of terbium iron garnet. Figure S6(b) presents the corrected HAADF image. Note that the image has been rotated for better visibility.

EELS intensity profiles

Figure S7 presents the EELS intensity profiles of Fe (black), Pt (red), and Al (blue) obtained from a line scan across the Pt(4 nm)/TbIG(2 nm)/Al:TbIG(23 nm) interface. The intensities are normalized to the maximum value for each element to enable comparison. The Pt signal (red) decreases sharply around 2–3 nm, marking the termination of the Pt layer, while the Fe signal (black) increases at the same position, corresponding to the onset of the TbIG film. The Al signal (blue) gradually increases within the TbIG layer, but its intensity remains relatively suppressed in the near-interface region of approximately 2 nm (between the two dashed green lines). We note that the origin of the position axis is not set at the top surface of the Pt(4 nm) layer. This result indicates that the interfacial 2-nm TbIG capping layer contains a lower Al concentration compared to the deeper part of the Al:TbIG layer, suggesting a relatively Fe-rich composition near the interface. Such a gradient of Al concentration at the capping layer is consistent with the stabilization of deterministic exchange bias discussed in the main text.

Quantifying relative atomic composition of Fe and Al by EELS

The relative elemental composition of Fe and Al was estimated using the Hartree–Slater cross-section model. The estimated relative composition from the Al and Fe edges yields 6–17% for Al (corresponding to 94–83% for Fe), depending on the choice of integration region and background subtraction window for each element. This range is reasonably compatible with the expected 5% Al content, considering that plural scattering was not corrected, the Hartree–Slater cross-section model provides only moderate accuracy, the Al signal is inherently weak, and beam propagation effects were not accounted for.

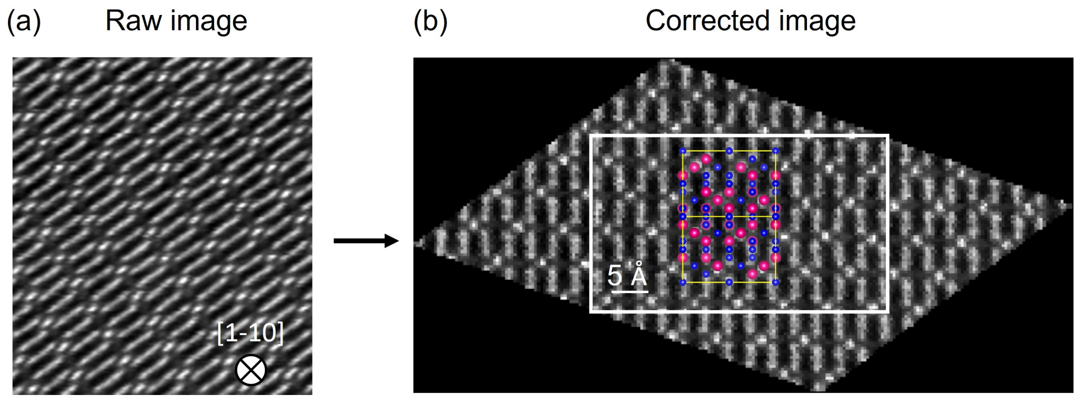

FIG. S6. (a) Raw HAADF image. (b) Corrected HAADF image. The garnet structure, indicating Fe (blue) and Tb (magenta) sites, is superimposed on the corrected image. Yellow lines denote the unit cell boundaries. The white boxed region is shown in the main text. The scale is calibrated based on the lattice constant of the sample (12.645 Å).

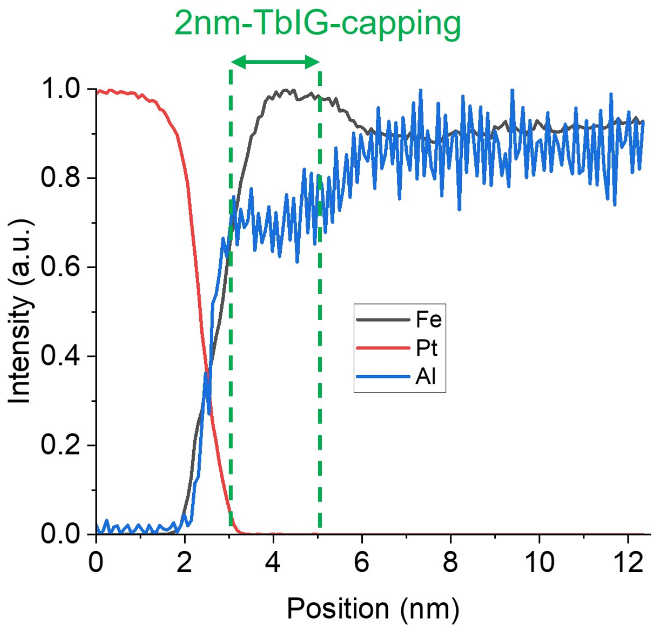

FIG. S7. EELS intensity profiles of Fe (black), Pt (red), and Al (blue) across the Pt(4 nm)/TbIG(2 nm)/Al:TbIG(23 nm) interface. The Al signal is suppressed in the near-interface region of the 2-nm TbIG capping layer. The green dashed lines indicate the approximate borders of the 2-nm TbIG capping layer.

S6. d-site preference of Al substitution

We estimated the preferential site of Al substitution between the a-site and d-site in the garnet structure using the EELS data. Figures S8(a) and (b) show the HAADF image and the corresponding EELS map of Al, respectively. A magnified view of the local area indicated by the square in Figures S8(a) is presented in Figures S8(c). The EELS intensity of Al was obtained at the positions labeled $d1$, $d2$, and a, averaged over several unit cells. Here, I_d and I_a denote the EELS intensities associated with the d- and a-sites, respectively. To calculate I_d, we summed the intensities from the two $d2$ windows and added half of the intensity from the $d1$ window. This normalization accounts for the fact that atomic columns at $d1$ are half as frequent as those at the $d2$ and a sites. This procedure yields an intensity ratio of $I_d : I_a \approx 2.53 : 1$. Accordingly, the probability that an Al atom occupies a d-site (a-site) is approximately 71% (29%). For comparison, if no preferential occupancy occurred, the probability would be 60% (40%), reflecting the 3:2 ratio of d- to a-sites in the crystal lattice. This result suggests a preferential occupation of Al at the d-site (tetrahedral oxygen coordination), which is consistent with the literature and with the observed increase of T_{M}.

This result should be interpreted with caution, since it neglects beam-propagation effects (such as dechannelling and channelling along the atomic columns of the oriented crystal, and the broadening of the electron probe as it goes through the lamella), which could either enhance or reduce the Al EELS signal at a given probe position. A reliable quantitative conclusion would require account for these effects in simulations, which in turn necessitate knowledge of the lamella thickness and the precise structure. Therefore, the present estimation should be regarded as a rough approximation of the Al site occupancy.

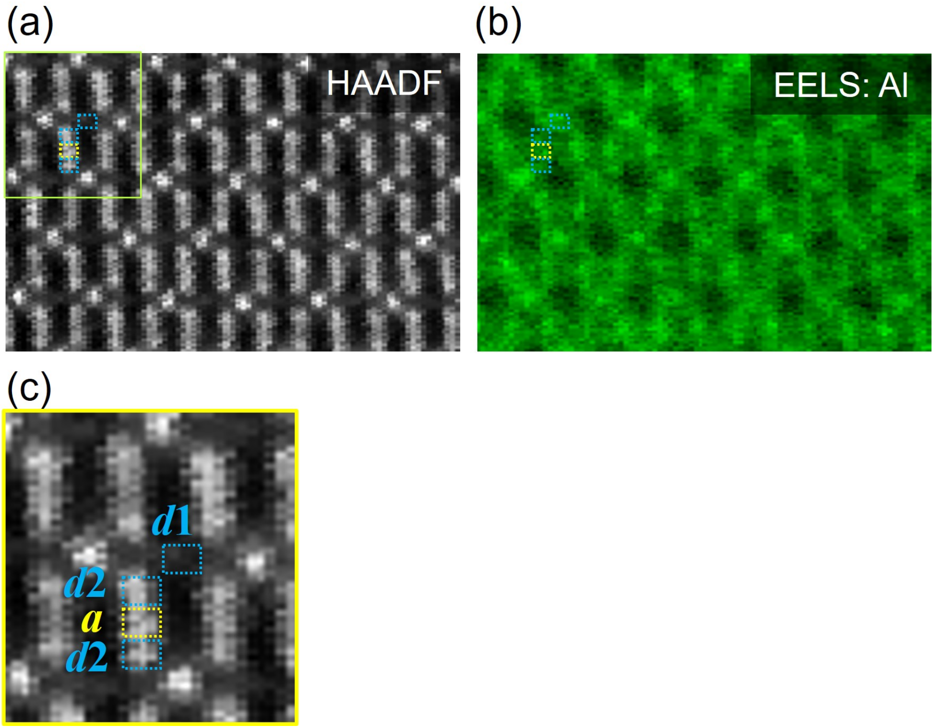

FIG. S8. (a) HAADF-STEM image of the Al-substituted TbIG film. (b) Corresponding EELS elemental map of Al. (c) Magnified HAADF image of the local area marked in (a), where the positions of the $d1$, $d2$, and a sites are indicated. The EELS intensities at these positions and other equivalent positions were used to estimate the preferential site occupancy of Al.

S7. Exchange bias in $P_{\mathrm{Al_2O_3}} = 0$ and 43 W

Figure S9 shows the Hall resistance R_H curves for (a) $P_{\mathrm{Al_2O_3}} = 0$ and (b) $P_{\mathrm{Al_2O_3}} = 43$ W ($T_\mathrm{M} = 215$ K), measured near the compensation temperatures. A sign reversal of the anomalous Hall effect (AHE) across T_M is observed in both cases, from which we determine $T_\mathrm{M} = 190$ K for $P_{\mathrm{Al_2O_3}} = 0$ and $T_\mathrm{M} = 215$ K for $P_{\mathrm{Al_2O_3}} = 43$ W. For $P_{\mathrm{Al_2O_3}} = 43$ W, a clear exchange bias (EB) is observed at 210 K, with $H_\mathrm{EB} = 3.8$ kOe. This suggests that the introduction of disorder into the system induces EB within a certain range of $P_{\mathrm{Al_2O_3}}$. Even in the undoped system ($P_{\mathrm{Al_2O_3}} = 0$), a small EB ($H_\mathrm{EB} = -0.48$ kOe) is observed at 185 K, which may be attributed to inherent magnetic site disorder arising from unavoidable defects in the sample. This result supports our T_M-distribution model for the emergence of EB.

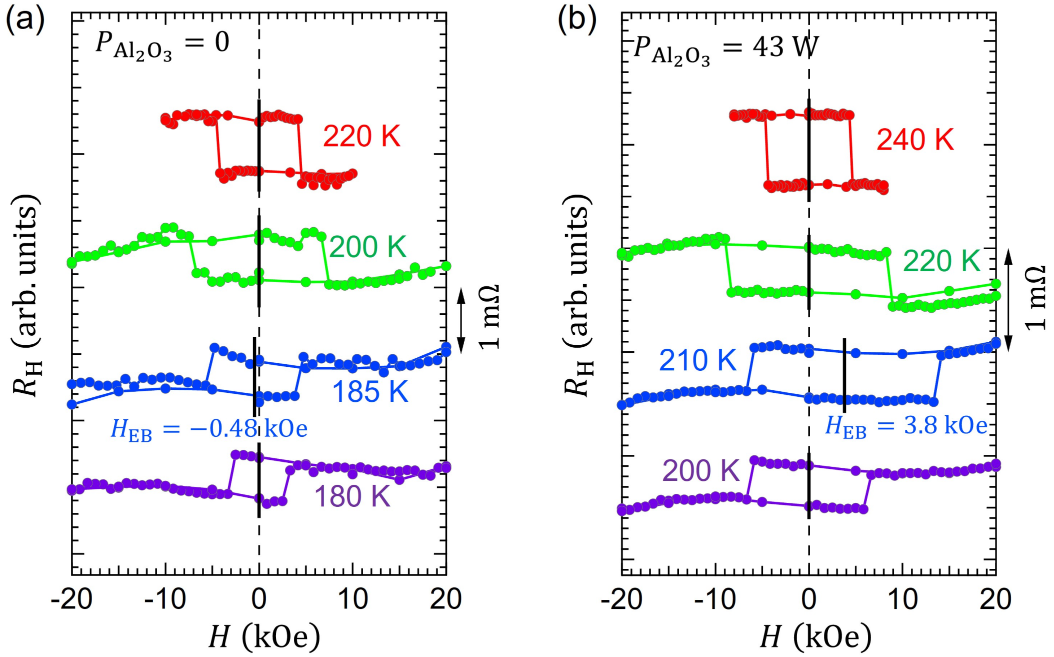

FIG. S9. R_H vs. H curves for (a) $P_{\mathrm{Al_2O_3}} = 0$ and (b) $P_{\mathrm{Al_2O_3}} = 43$ W measured near the compensation temperatures. The linear contribution from the ordinary Hall effect has been subtracted from the data.

S8. Examination of ZFC stochastic exchange bias in different devices

In the main text, we presented results demonstrating stochastic exchange bias behavior (after ZFC) for three different devices (labeled Dev-1 to Dev-3) on the substrate (for $P_{Al_2O_3} = 65$ W). Our thin film sample contains 16 devices on the substrate, with their positions indicated schematically and labeled (e.g., "A1") in the bottom-right corner of Fig. S10. To assess the reproducibility of the stochastic exchange bias behavior, we examined six additional devices.

In Fig. S10, we plot H_{c1} and H_{c2} as functions of the cycle number (n) for several devices, including Dev-1 to Dev-3. Among the additional devices tested, two (C1 and B2) also exhibited stochastic behavior (highlighted in green), whereas the other four did not show such stochastic features (highlighted in red). Nevertheless, all the devices measured here exhibited exchange bias.

It appears that devices located closer to the center of the substrate are more likely to exhibit stochastic behavior. This observation suggests that the stochastic nature may be sensitive to growth conditions, which could vary slightly depending on the position on the substrate (e.g., near the center vs. the outer edges).

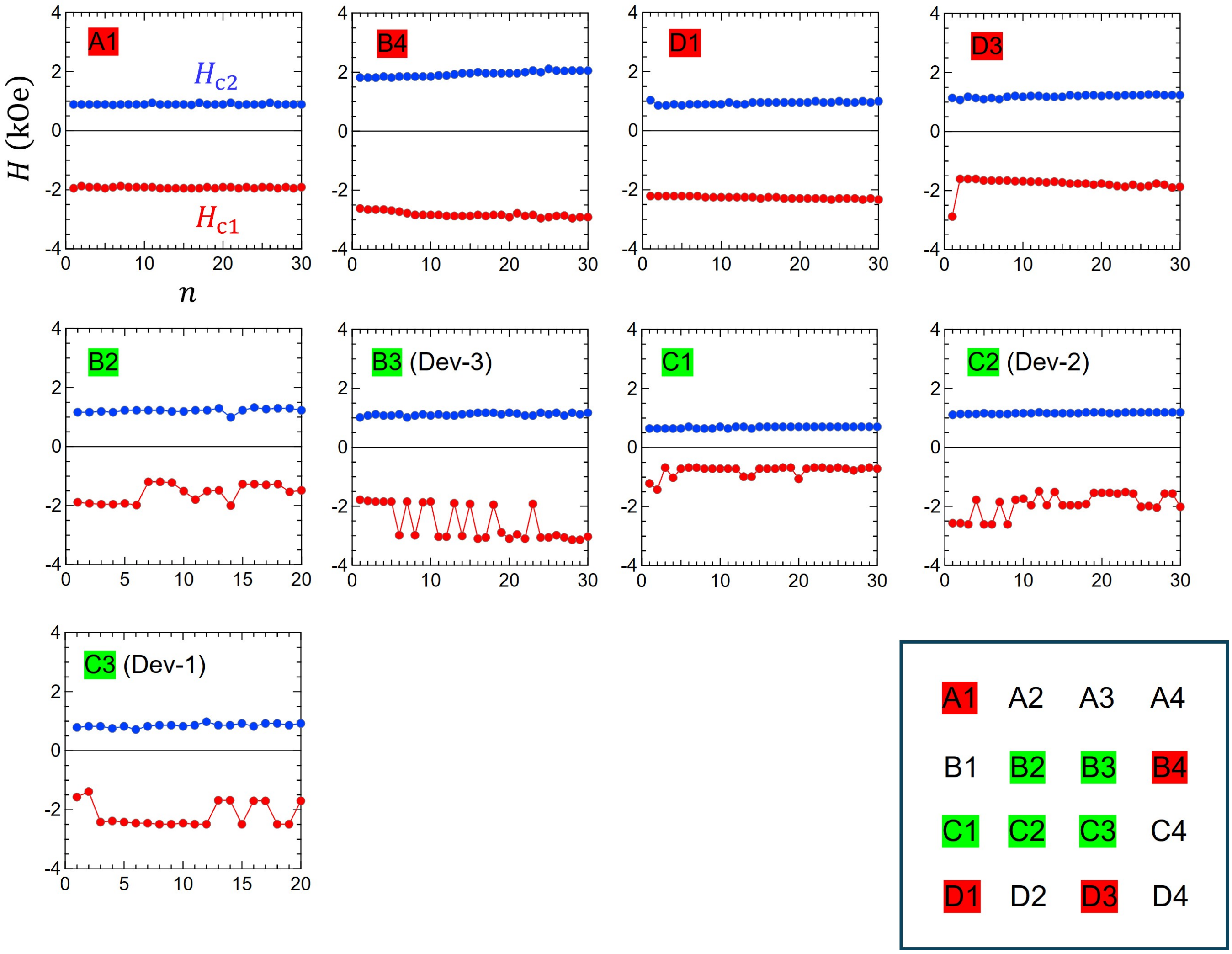

FIG. S10. The plots of H_{c1} and H_{c2} as a function of the cycle number (n) for different devices. The schematic in the right bottom indicates the positions of the devices. The color on the labels indicates the presence (green) or absence (red) of the stochastic exchange bias.

S9. ZFC stochastic exchange bias for the initial negative bias case

Figure S11(a) shows twenty AHE hysteresis loops measured sequentially at 297 K after zero-field cooling (ZFC) for the negative-bias case, where the initial field sweep ($n = 1$) started from a negative field of $H \approx -4$ kOe. In contrast to the positive-bias case (started from a positive field), the direction of the stochastic exchange bias (EB) is reversed. For the negative-bias case, H_{c2} varies stochastically, whereas H_{c1} remains fixed (see Fig. S11(b)).

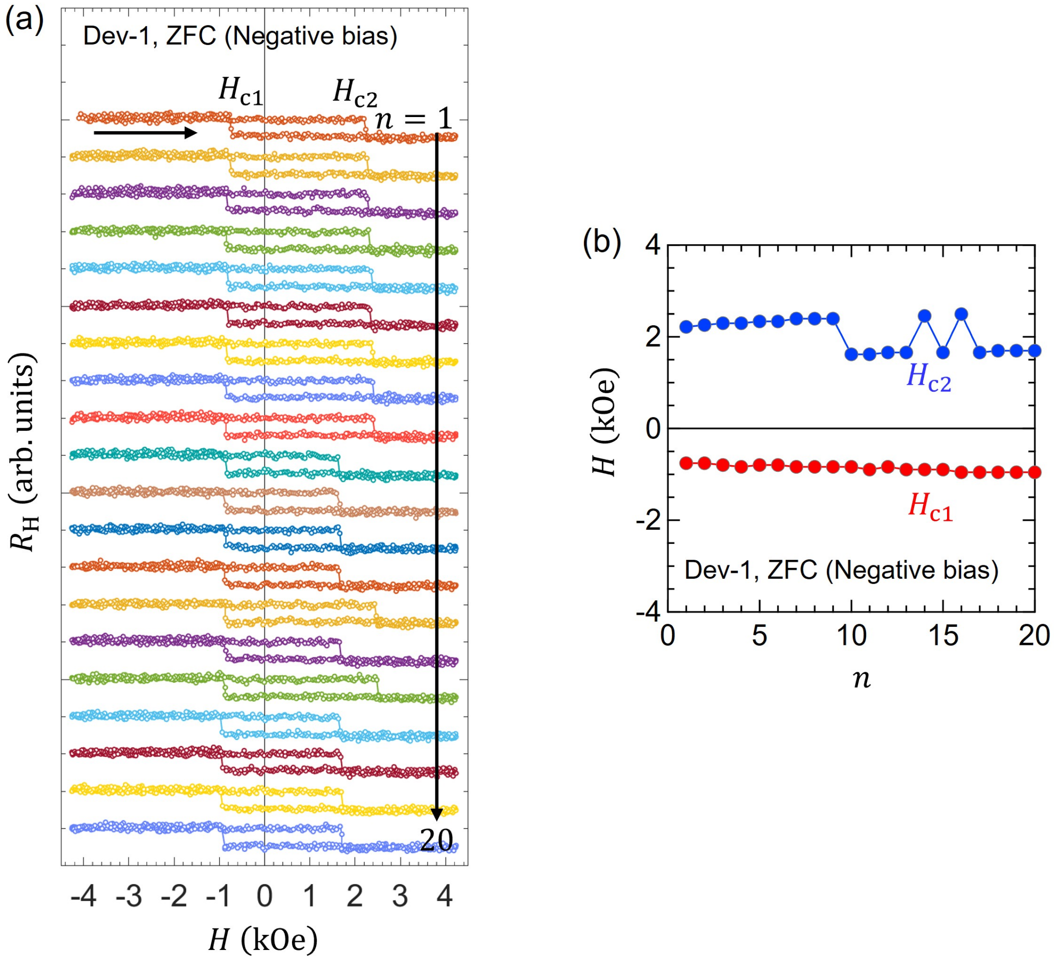

FIG. S11. (a) Twenty AHE hysteresis loops measured at 297 K in sequence after zero field cooling (ZFC) for the initial negative-bias case. Note that linear slopes from ordinary Hall effect component are subtracted. (b) The plot of H_{c1} and H_{c2} as a function of the cycle number (n).

S10. Derivation of the temperature dependence of exchange bias anisotropy

We derive the expectation value of unidirectional EB anisotropy U considering the distribution ($n(T_{\rm M}^{\rm loc})$) of the local magnetic compensation temperature $T_{\rm M}^{\rm loc}$. Here we assume a Gaussian type distribution, but the following calculation is independent of the details of the distribution function. To simplify the calculation, we discretize $n(T_{\rm M}^{\rm loc})$ considering N possible discrete values of $T_{\rm M}^{\rm loc}$ denoted as T_i $(i = 1, 2, \cdots, N)$ (see Fig. S12).

The fraction of the local states that exhibit $T_{\rm M}^{\rm loc} = T_i$ can be described as follows:

$$n(T_i) = \frac{G(T_i)}{Z}, \tag{4}$$

where $G(T) \equiv \exp\left[-\frac{(T-T_{\rm M}^{*})^2}{2\sigma^2}\right]$ is a Gaussian-type function, and Z (giving the normalization factor) is defined as follows:

$$Z \equiv \sum_{i}^{N} G(T_i). \tag{5}$$

Obviously, Eq. 4 satisfies the following normalization:

$$\sum_{i}^{N} n(T_i) = 1. \tag{6}$$

We consider that there are $N_{\rm domain}$ local domains. When the neighboring local domains have the pair of ferromagnetic (FM) and antiferromagnetic (AFM) states, a local EB state can be expected. The expectation value of the unidirectional (EB) anisotropy energy at T_i over the system (consisting of $N_{\rm domain}$ local domains) should be given as follows:

$$U(T_i) = \sum_{<j,k>} u^{(jk)} p_j(T_i)[1 - p_k(T_i)], \tag{7}$$

where $p_j(T_i)$ $(0 \leq p_j(T_i) \leq 1)$ is the probability that the site j at T_i is in the AFM state, while $[1 - p_k(T_i)]$ represents the probability that the site k at T_i is in the FM state. The coefficient $u^{(jk)}$ represents the magnitude of local EB anisotropy.

Here we consider the large number of local domains and possible $T_{\rm M}^{\rm loc}$ values (i.e., $N_{\rm domain} \gg 1$ and $N \gg 1$), and the following replacement should be valid: $p_j(T_i) \to n(T_i)$. Eq. 7 can be written as follows

$$U(T_i) = n(T_i)[1 - n(T_i)] \sum_{<j,k>} u^{(jk)} = U_0^{'} n(T_i)[1 - n(T_i)], \tag{8}$$

where $U_0^{'} \equiv \sum_{<j,k>} u^{(jk)}$ is a constant. Inserting Eq. 4 into Eq. 8, we obtain

$$U(T_i) = U_0^{'} \frac{G(T_i)}{Z} \left[1 - \frac{G(T_i)}{Z}\right]. \tag{9}$$

The value of Z and $U_0^{'}$ increases with the value of N and $N_{\rm domain}$, respectively: namely, $Z \sim N$ and $U_0^{'} \sim N_{\rm domain}$. Regarding the order of the magnitude of N and $N_{\rm domain}$, it is reasonable to assume $N \sim N_{\rm domain}$. Thus the orders of magnitudes of Z and $U_0^{'}$ are also the same (i.e., $Z \sim U_0^{'} \sim N$). By redefining $U_0 \equiv U_0^{'}/Z$ $(U_0 \ll N)$ and considering that $U_0/Z \sim U_0/N \to 0$ for $N \gg 1$ (thus the second term of the right side of Eq. 9 drops), Eq. 9 can be simplified as follows:

$$U(T_i) \approx U_0 G(T_i). \tag{10}$$

In the large N limit, T_i can be replaced by a continuous variable $(T_i \to T)$. Thus we obtain

$$U(T) \approx U_0 G(T). \tag{11}$$

This equation tells that the unidirectional EB anisotropy energy has the same temperature dependence as the distribution of $T_{\rm M}^{\rm loc}$.

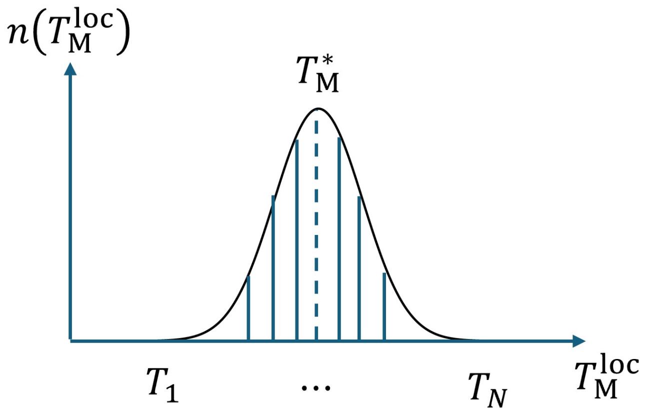

FIG. S12. Distribution of discritized local compensation temperature T_i $(i = 1, 2, \cdots, N)$.

S11. Validity of Gaussian distribution

We examine the temperature dependence of $U(T)$ from the experimental data in detail and assess the validity of the Gaussian distribution [Eq. (1) in the main text]. In a narrow temperature range near $T_{\rm M}^*$ (and well below the Curie temperature), the uniaxial anisotropy K and the Fe-sublattice magnetization $M_{\rm Fe}$ can be assumed to be constant (i.e., $K =$ const. and $M_{\rm Fe} =$ const.), whereas $U(T)$ should be temperature-dependent since EB was observed only in the vicinity of $T_{\rm M}^*$. Given that $H_{\rm c} \propto K$ and $H_{\rm EB} \propto U$, the temperature dependence of these quantities (around $T_{\rm M}^*$) can be extracted from Eq. (2) in the main text as follows:

$$H_{\rm c}(T) = \frac{K}{2M_{\rm Fe}} F(T), \text{ and } H_{\rm EB}(T) = -\frac{U(T)}{2M_{\rm Fe}} F(T), \tag{12}$$

where $F(T) \equiv (T - \theta_{\rm w})/(T - T_{\rm M}^*)$. Note that the temperature dependence of $H_{\rm c}$ is described by $F(T)$, whereas that of $H_{\rm EB}$ is determined by the product of $U(T)$ and $F(T)$.

The objective here is to analyze $U(T)$ based on the experimental data of $H_{\rm EB}(T)$. To achieve this, we plot the experimental data for $H_{\rm c}$ and $H_{\rm EB}$ as functions of $F(T)$. In Fig. S13(a), we show the data points indicated by the triangle symbols in Fig. 4(c) in the main text. These data points were systematically obtained after a single FC process, where the significant stochastic variation of EB is absent. As expected, $H_{\rm c}$ exhibits good linearity, where the slope provides the value of $K/(2M_{\rm Fe})$. The solid linear line represents the model with $K/(2M_{\rm Fe}) = 18$ Oe. On the other hand, $H_{\rm EB}$ (plotted as $|H_{\rm EB}|$) exhibits a nonlinear behavior, indicating that $U(T)$ indeed has a temperature dependence. The continuous trend observed in the experimental data indicates that $U(T)$ is a continuous function. The solid-line curve represents the model based on the Gaussian function of $U(T)$ given by Eq. (1) in the main text, with $U_0/(2M_{\rm Fe}) = 12$ Oe and $\sigma = 4$ K. Remarkably, the model curve with the Gaussian distribution accurately describes the nonlinear behavior of the experimental $H_{\rm EB}$ data, supporting the validity of the Gaussian function of $U(T)$.

The onset of the increase in $H_{\rm EB}$ corresponds to the edge of the distribution. The black arrow labeled as 3σ indicates the value of $F(T)$ for $T = T_{\rm M}^* + 3\sigma$ (where $\sigma = 4$ K). Note that, in a Gaussian distribution, 99.7% of the data lie within the ranges of $\pm 3\sigma$. Since 3σ provides an approximate estimate of the edge of the distribution, the onset of $H_{\rm EB}$ allows us to experimentally determine the value of σ. Thus, plotting $H_{\rm EB}$ as a function of $F(T)$ proves to be a useful approach for characterizing the distribution function $U(T)$ experimentally if there is no stochastic variation in $H_{\rm EB}$.

We note that the model parameters determined here [$K/(2M_{\rm Fe}) = 18$ Oe, $U_0/(2M_{\rm Fe}) = 12$ Oe, $\sigma = 4$ K] differ from those used in the main text [$K/(2M_{\rm Fe}) = 30$ Oe, $U_0/(2M_{\rm Fe}) = 15$ Oe, $\sigma = 10$ K]. This is because the parameters in the main text were determined by accounting for the stochastic variation of EB states arising from different FC processes, in order to reproduce the overall distribution of the experimental data points.

The Gaussian distribution, as a standard statistical distribution, is a natural choice from a statistical point of view. Nevertheless, for comparison, we tested several alternative distribution functions. Figures S13(b)–(d) show plots of $H_{\rm EB}$ as a function of $F(T)$ for (b) a uniform distribution, (c) an exponential distribution, and (d) a Cauchy distribution. The corresponding model curves and equations are shown in the upper panels, and the parameter values used for each plot are indicated within the figures. As seen in Fig. S13(b), the uniform distribution—being discontinuous—clearly fails to reproduce the smooth variation of $H_{\rm EB}$. Among the tested models, the Gaussian distribution provides the best fit to the experimental data. However, we note that other continuous distributions (e.g., the Cauchy distribution) cannot be definitively ruled out given the accuracy of our measurements. Note that the exponential distribution gives a reasonable fit only when a relatively large value of $U_0/K = 1.2$ (i.e., $U_0 > K$) is assumed, which appears to be unrealistic in the context of our system. In summary, there is no compelling reason to prefer any of the alternative distributions over the standard Gaussian form. What is essential is that the distribution function is continuous and possess a finite width.

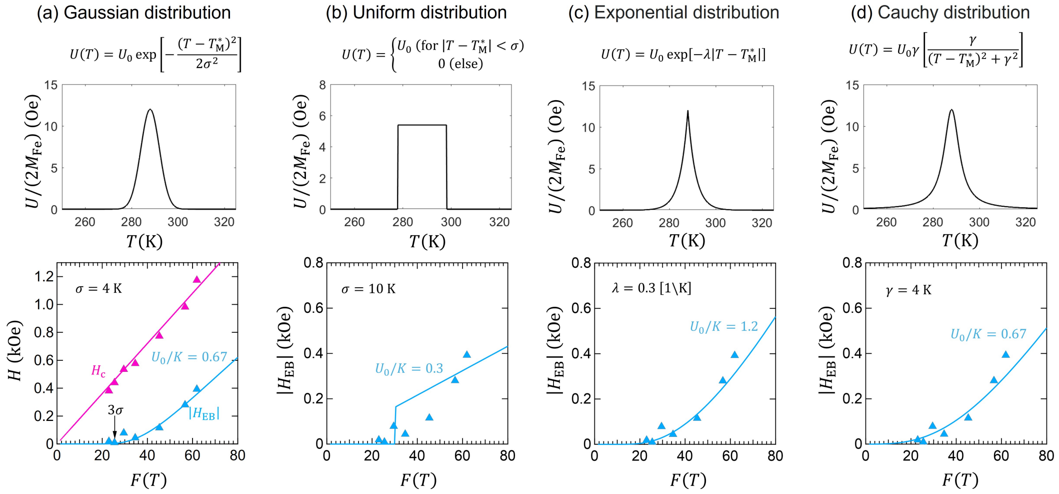

FIG. S13. Validity checks for several distribution models: (a) Gaussian distribution, (b) uniform distribution, (c) exponential distribution, and (d) Cauchy distribution. The upper panels show the model distribution curves. The lower panels plot $H_{\rm EB}$ as a function of $F(T) = (T - \theta_{\rm w})/(T - T_{\rm M}^{*})$, with $\theta_{\rm w} = -7$ K and $T_{\rm M}^{*} = 288$ K. The triangle symbols represent the experimental data, whereas the solid lines indicate the model curves. See the main text for the black arrow labeled 3σ. In panel (a), the coercive field $H_{\rm c}$ is also plotted for comparison (shown as magenta symbols and line).

S12. Possible explanation for the unidirectional nature of the stochastic EB

The ZFC stochastic EB behavior during the field sweeps (and the variation of switching field values for different FC processes) appears only in the hard-switching side (H_{c1} for $T > T_M$). We discuss the cause of this unidirectional nature of the stochastic EB using our T_M^{loc} distribution model. As discussed in the main text, it is expected that the Gaussian distribution profile may change during the field sweep measurement for the ZFC case (and also may change depending on the FC processes).

We explore how the switching field curves change depending on the change in the distribution function. We rewrite Eq. (1) in the main text, which is the Gaussian distribution of unidirectional EB anisotropy energy (U):

$$U(T) \approx U_0 \exp\left[-\frac{(T - T_M^*)^2}{2\sigma^2}\right], \tag{13}$$

where we set $\sigma = 10$ K. Figure S14(a) displays the plot of Eq. 13 (plotted as $U/(2M_{Fe})$) for $U_0/(2M_{Fe}) = 15$ Oe. Here we plotted curves for different values of T_M^* in the range of $[T_{M,0}^* - \delta T_M^*, T_{M,0}^* + \delta T_M^*]$, where we set the values of $T_{M,0}^* = 288$ K and $\delta T_M^* = 3$ K. For each U profile, we calculated the switching fields (H_{c1} and H_{c2}) using Eq. (2) in the main text (with $K/(2M_{Fe}) = 30$ Oe). Figures S14(b)-(e) display the variation of switching fields depending on the value of T_M^* for $U_0/(2M_{Fe}) = 9$, 15, 21 and 27 Oe, respectively. At the measurement temperature 297 K (indicated by the vertical solid line), it can be seen that the variation of H_{c2} is less than that of H_{c1}. Figure S14(f) shows the U_0 dependence of the variations ΔH_{c1} and ΔH_{c2} at $T = 297$ K, which are defined as $\Delta H_{c1} \equiv |H_{c1}(T_{M,0}^* + \delta T_M^*) - H_{c1}(T_{M,0}^* - \delta T_M^*)|$ and $\Delta H_{c2} \equiv |H_{c2}(T_{M,0}^* + \delta T_M^*) - H_{c2}(T_{M,0}^* - \delta T_M^*)|$. The difference between ΔH_{c1} and ΔH_{c2} becomes more pronounced as the value of U_0 increases, leading to the asymmetric unidirectional nature of the variation of switching fields. This could be a possible explanation for the experimentally observed unidirectional stochastic behavior. Here we demonstrated one of the possible changes in the distribution function, but the other changes in the distribution function (e.g., the shape of the distribution profile and the value of σ) could also lead to a similar result.

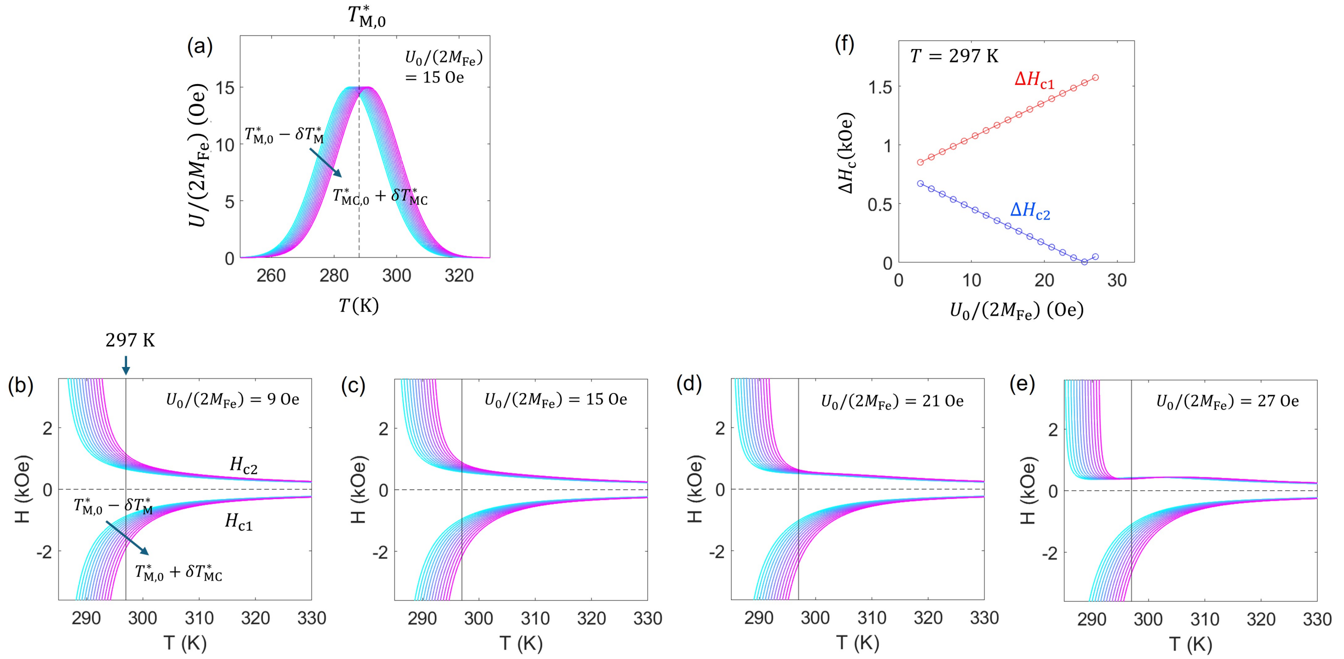

FIG. S14. (a) The Gaussian distribution function (plotted as $U/(2M_{Fe})$) as a function of temperature for different values of T_M^*. (b)-(e) The variation of switching fields H_{c1} and H_{c2} (calculated using Eq. (2) in the main text) depending on the value of T_M^* for $U_0/(2M_{Fe})$ = (b) 9, (c) 15, (d) 21 and (e) 27 Oe, respectively. The vertical lines indicate the field-sweep measurement temperature (297 K). (f) The U_0 dependence of the variations ΔH_{c1} and ΔH_{c2} at $T = 297$ K.

* tshiino@icmab.es
† cavci@icmab.es

[1] H. Fuess, G. Bassi, M. Bonnet, and A. Delapalme, Neutron scattering length of terbium structure refinement and magnetic moments of terbium iron garnet, Solid State Communications **18**, 557 (1976).
[2] H. Chikh, F. S. Ahmed, A. Afir, and A. Pialoux, In-situ X-ray diffraction study of alumina α-Al2O3 thermal behavior under dynamic vacuum and constant flow of nitrogen, Journal of Alloys and Compounds **654**, 509 (2016).
[3] S. Vural and Özlem Sari, Synthesis and characterization of SDS assistant α-alumina structures and investigation of the effect of the calcination time on the morphology, Colloid and Polymer Science **297**, 107 (2019).
